# 21st‐Century Mangrove Expansion Along the Southeastern United States

**DOI:** 10.1111/gcb.70676

**Published:** 2026-01-07

**Authors:** Lucia I. A. Enes Gramoso, Dustin Carroll, Kyle C. Cavanaugh, Remi Bardou, Michael J. Osland, Tom Van der Stocken

**Affiliations:** ^1^ Department of Biology, bDIV: Ecology, Evolution & Genetics Vrije Universiteit Brussel Brussels Belgium; ^2^ Moss Landing Marine Laboratories San José State University Moss Landing California USA; ^3^ Earth Science Section, Jet Propulsion Laboratory California Institute of Technology Pasadena California USA; ^4^ Department of Geography University of California Los Angeles California USA; ^5^ Institute for Global Change Biology University of Michigan Ann Arbor Michigan USA; ^6^ U.S. Geological Survey Lafayette Louisiana USA

**Keywords:** climate change, coastal wetland, ecosystem services, habitat suitability modeling, Lagrangian particle‐tracking, ocean dispersal, range expansion

## Abstract

Warming winter temperatures are driving range expansion of tropical, cold‐sensitive mangroves into temperate ecosystems. Along the Atlantic coast of North America, the mangrove range limit is particularly sensitive to climate variability and historical data demonstrate that the mangrove‐salt marsh ecotone on this coast has shifted recurrently during recent centuries. However, a comprehensive understanding of how this mangrove‐salt marsh ecotone may shift in the future remains lacking. Here, we combine ensemble forecasting of mangrove distribution for the next century with high‐resolution oceanographic dispersal simulations, phenological observations, and historical hurricane data to project future mangrove‐salt marsh dynamics at the rapidly changing range limit in northeastern Florida (USA). We show that warming winter temperatures will drive continued poleward expansion of mangroves along North America's Atlantic coast, potentially reaching South Carolina by 2100. With ongoing climate change, suitable mangrove habitat is projected to expand beyond the current range limit, and dispersal simulations suggest successful colonization of these sites from established mangrove populations. Additionally, patterns in hurricane directionality and intensity and field reports of propagule presence reveal that these high‐energy events may significantly contribute to future mangrove range expansion by facilitating long‐distance, storm‐driven propagule dispersal. The encroachment of mangroves in salt marsh‐dominated latitudes is expected to substantially modify wetland ecosystem function and structure, emphasizing how the identification of newly colonizable habitat can inform conservation strategies and site‐specific decisions on mangrove management.

## Graphical abstract

Global warming is driving poleward range shifts worldwide, including the northward expansion of tropical mangroves along the Atlantic coast of North America. In this region, warming winters are enabling mangroves to encroach into salt marsh–dominated temperate latitudes, potentially transforming wetland ecosystem function and services. To assess future shifts in the mangrove–salt marsh ecotone, we projected mangrove habitat suitability under four IPCC SSP scenarios (2071–2100) and combined these projections with high‐resolution oceanographic dispersal simulations, phenological observations, and historical hurricane data to determine whether mangrove propagules can reach newly suitable habitats. Our results indicate that warming winter temperatures are likely to drive continued poleward expansion of mangroves along North America's Atlantic coast, potentially reaching South Carolina by 2100, facilitated by both oceanic and hurricane‐driven propagule transport from established populations. These findings are key for guiding conservation and wetland management decisions.

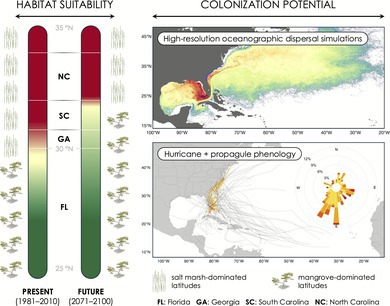

## 1 Introduction

Climate change is driving range shifts of numerous marine, freshwater, and terrestrial organisms (Rosenzweig et al. 2008; Chen et al. 2011; Lenoir and Svenning 2015), which is altering the structure and function of ecosystems globally (Hoegh‐Guldberg and Bruno 2010; Nolan et al. 2018). Along the Atlantic coast of Florida (USA), a poleward expansion of mangroves occurs as a threshold response to warmer winters with fewer extreme freeze events, facilitating encroachment of tropical mangroves into temperate salt marshes (Saintilan et al. 2014; Cavanaugh et al. 2014; Sheridan and Lee 2018; Vervaeke et al. 2025). Previous research has shown that the mangrove‐salt marsh ecotone on this coast has shifted recurrently during recent centuries due to natural climate variability, but that the recent mangrove expansion may represent a more permanent regime shift due to anthropogenic climate change (Cavanaugh et al. 2019; Bardou et al. 2021). Understanding the drivers of mangrove expansion into salt marshes can be used in predicting future mangrove‐salt marsh ecotone dynamics and anticipating the ecological and societal implications of these ecosystem transformations. However, previous studies have focused primarily on the role of macroclimatic variables without explicitly quantifying the probability of mangroves colonizing newly suitable habitats via ocean dispersal. Filling this knowledge gap is relevant, as mangroves and salt marshes provide diverse ecosystem services, including carbon sequestration, coastal protection, erosion control, and nutrient cycling (Walters et al. 2008; Barbier et al. 2011). Potential future ecological transitions, from herbaceous or succulent salt marsh to woody mangroves, could substantially alter the function and socioeconomic services provided by these intertidal wetlands (Osland et al. 2013, 2022; Doughty et al. 2016; Kelleway et al. 2017; Vaughn et al. 2020).

Mangroves comprise a group of coastal wetland plants that grow on sheltered shorelines in tropical, subtropical, and some warm temperate latitudes (Spalding et al. 2010). Living at the land–sea interface, mangroves have evolved a range of morphological and physiological traits to thrive in this dynamic environment (Tomlinson 2016). Species in most genera produce viviparous propagules that can disperse via tidal, alongshore, and open‐ocean currents over local‐to‐transoceanic scales (Van der Stocken, Carroll, et al. 2019; Van der Stocken, Wee, et al. 2019). Processes that influence the dispersal of mangrove propagules over these spatial scales play an important role in controlling climate‐change‐related range expansion and ecotone shifts (Nathan et al. 2008). Along the east coast of Florida, dispersal and establishment of propagules during freeze‐free winters allow mangrove forests to regenerate and expand beyond their range limit, converting coastal wetlands to a mangrove‐dominated state (Cavanaugh et al. 2014, 2019). Additionally, high‐energy tropical storm events—such as the hurricanes that regularly affect Florida—can facilitate dispersal beyond the most‐northern established populations (Lugo 2008; Kennedy et al. 2020).

In this study, we combine ensemble forecasting of mangrove distribution, Lagrangian particle‐tracking, hurricane data, and reported phenological (i.e., timing of propagule development and release) data to project future mangrove expansion at the rapidly changing range limit in northeastern Florida. We develop species distribution models (SDMs) to identify suitable mangrove habitat, both within and beyond the current mangrove distribution, under present and future climate scenarios. Correlative SDMs relate geographic occurrences with a set of predictor variables to estimate the ecological requirements of species (Araújo and Guisan 2006). However, assuming species distributions are solely determined by climatic factors is often an oversimplification. For example, regional studies indicate that other drivers, such as dispersal limitation, can control the position of certain range boundaries (Cavanaugh et al. 2024; Osland et al. 2017; Raw et al. 2023). We therefore use a high‐resolution, eddy‐ and tide‐resolving numerical ocean model with hourly releases to simulate individual mangrove propagule trajectories. This model allows us to examine the spatial scales of simulated propagule transport in the region, test whether dispersal beyond the present‐day mangrove range limit is likely, and assess connectivity between established mangrove populations and unoccupied present and future suitable mangrove habitat. Finally, hurricane data spanning 1851–2023 are used to study patterns in the directionality and intensity of tropical storms and interpret these patterns against phenological observations.

## 2 Results

### 2.1 Species Distribution Modeling

Six species distribution models were generated to predict the distribution of mangroves along the southeastern Atlantic coast of North America. All six were incorporated into the ensemble model, as they showed high values across the evaluation metrics (Table 1). The overall accuracy (ACC) ranged from 0.859 to 0.893, the area under the receiver operating characteristics curve (AUC) from 0.911 to 0.955, and the true skill statistics (TSS) from 0.802 to 0.842. The combined output of these models provides probabilities for mangrove occurrence across the study region. The habitat suitability threshold to assess whether locations can sustain mangrove growth was set at 0.54, as the mean threshold yielded higher values for model sensitivity, TSS, kappa, and AUC (Table 2).

**TABLE 1:** Model evaluation metrics: Overall accuracy (ACC), area under the receiver operating characteristic curve (AUC), and true skill statistic (TSS) are reported for the six distribution models.

	CTA	FDA	GAM	GBM	GLM	RF
ACC	0.859	0.875	0.876	0.892	0.876	0.893
AUC	0.911	0.944	0.938	0.955	0.942	0.952
TSS	0.823	0.822	0.802	0.842	0.823	0.842

Abbreviations: CTA, classification tree analysis; FDA, flexible discriminant analysis; GAM, generalized additive model; GBM, boosted regression trees; GLM, generalized linear model; RF, random forest.

**TABLE 2:** Threshold‐dependent statistics: Model sensitivity, model specificity, Cohen's kappa statistic, area under the receiver operating characteristic curve (AUC), true skill statistic (TSS), and overall accuracy (ACC) for the median, conservative, and mean probability thresholds.

	Value	Sensitivity	Specificity	TSS	Kappa	ACC	AUC
Mean	0.539	0.955	0.869	0.824	0.691	0.885	0.912
Median	0.610	0.890	0.891	0.780	0.689	0.890	0.890
Conservative	0.750	0.691	0.939	0.631	0.644	0.892	0.815

**FIGURE 1:**
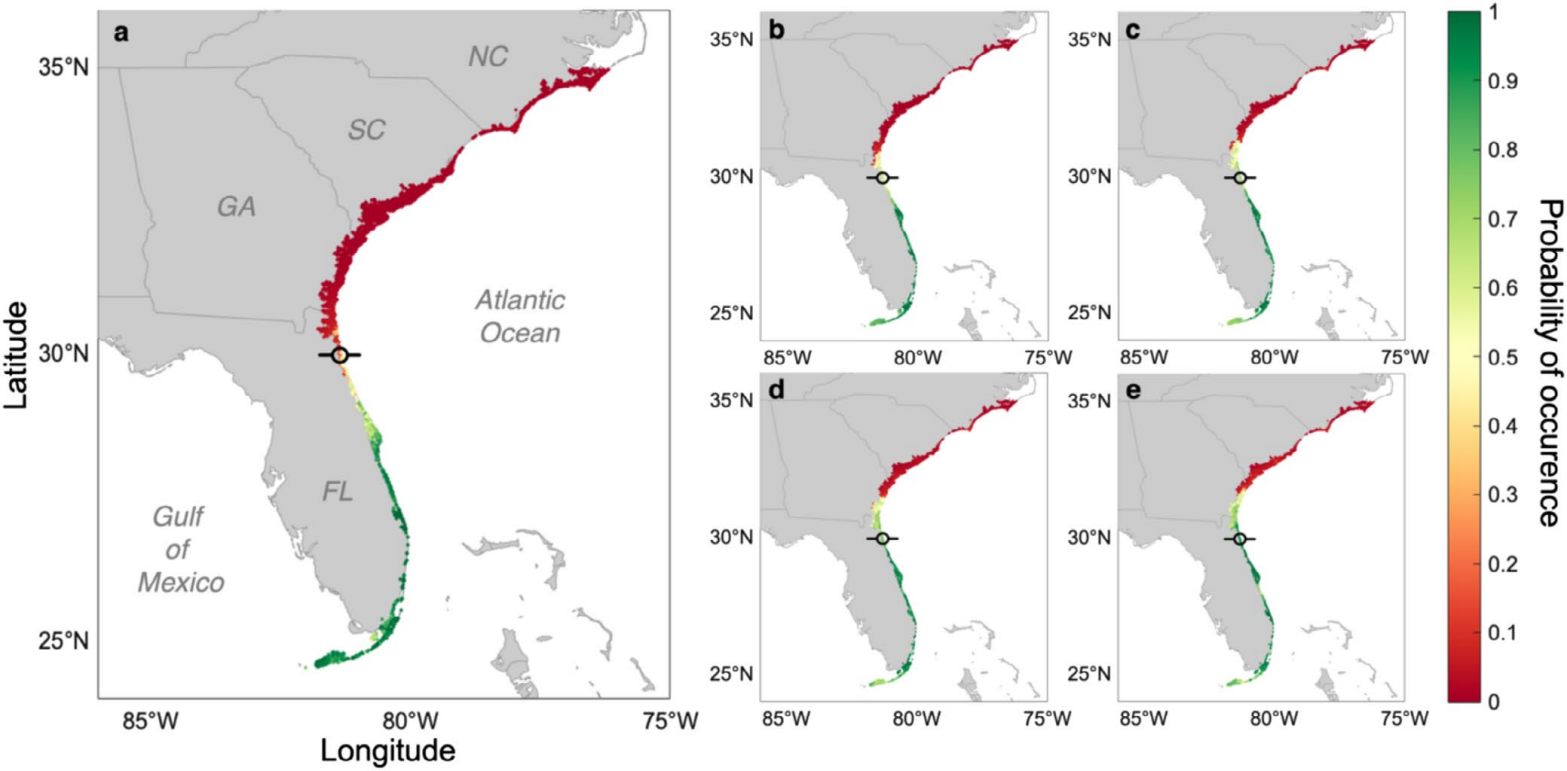
Mean probability of mangrove occurrence along the Atlantic coast of the United States under (a) present (1981–2010) conditions and future (2071–2100) climate scenarios: (b) SSP1‐2.6, (c) SSP2‐4.5, (d) SSP3‐7.0, (e) SSP5‐8.5. Probability of occurrence is only provided for points in our study area containing either mangrove or salt marsh data. The current mangrove distribution limit in the FDEP dataset is displayed in each map as a circle with a horizontal bar. FL: Florida, GA: Georgia, SC: South Carolina, and NC: North Carolina. Note the changes across the northern FL and GA coasts. Map lines delineate study areas and do not necessarily depict accepted national boundaries.

We validate our ensemble model by comparing the present‐day mangrove distribution predictions (Figure 1a) with known presence–absence data. The model predicted the most poleward suitable location under present conditions at 29.74° N, which closely aligns with the present‐day position of the mangrove range limit in the Florida Department of Environmental Protection (FDEP) 2022 mangrove occurrence data (29.97° N). Species distribution projections for the period 2071–2100 predicted a poleward expansion of the habitat suitable for mangrove occurrence for all four Shared Socio‐Economic Pathways (SSP1‐2.6; SSP2‐4.5; SSP3‐7.0; SSP5‐8.5; Figure 1b–e). All four future scenario predictions indicate suitable mangrove habitat extending beyond the current range limit, with the extent of suitable mangrove habitat increasing under more severe climate change scenarios (i.e., from SSP1‐2.6 to SSP5‐8.5). The northernmost suitable locations are predicted at 30.72° N, 31.29° N, 31.45° N, and 31.76° N under SSP1‐2.6, SSP2‐4.5, SSP3‐7.0, and SSP5‐8.5, respectively. Under the low‐emissions scenario (SSP1‐2.6), 76% of wetland locations in east Florida are predicted to be suitable for mangrove occurrence, which increases to 85% under the high emissions scenario (SSP5‐8.5). Suitable habitat is projected to extend as far north as Georgia, where 44% of the coastal wetland locations are predicted to exceed the habitat suitability threshold under SSP5‐8.5. For this climate scenario, non‐zero probabilities are found as far north as South Carolina (Figure 1e).

### 2.2 Simulated Propagule Transport

**FIGURE 2:**
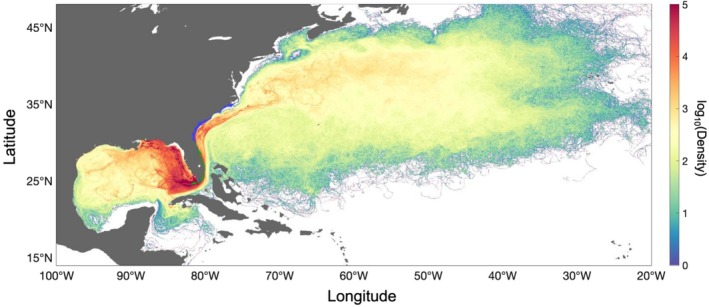
Simulated trajectory density map of mangrove propagules released along the Atlantic coast of Florida. Propagules were released hourly over a three‐month period (August–October) with a maximum floating period of 17 months. Dispersal trajectories were aggregated on a 1/24° × 1/24° grid. Green and blue points along the Atlantic coast of Florida indicate present‐day mangrove and salt marsh occurrence, respectively, with particles released from present‐day mangrove locations (green). Map lines delineate study areas and do not necessarily depict accepted national boundaries.

For all simulated propagule floating periods, high propagule trajectory densities are found along the southeastern coast of North America, corresponding to the region with release locations and in the eastern Gulf coast along the west Florida shelf (Figure 2). The north/northeastern transport, following the Florida Current into the North Atlantic Ocean, exhibited a dominant along‐shore component, while propagules transported over the west Florida shelf tend to concentrate near the southern tip of the shelf or travel northwestwards into the Gulf of Mexico. Although high propagule trajectory densities were observed along the west Florida coast, ocean currents did not allow for dispersal toward the transition zone between the Florida panhandle and the Florida peninsula. Particle transport simulations with maximum floating periods of 6, 12, and 17 months show propagule dispersal to coastal regions within the Gulf of Mexico (Appendix S1, Figures S25 and S26). For simulations of 12 and 17 months, propagules stranded along the coast of Texas, Louisiana, and West Florida, with occasional dispersal events to Mexico. For shorter floating times of 1 and 3 months, propagules only stranded along the East Coast of the United States, with no dispersal in the Gulf of Mexico (Appendix S1, Figures S23 and S24).

### 2.3 Connectivity Matrices

**FIGURE 3:**
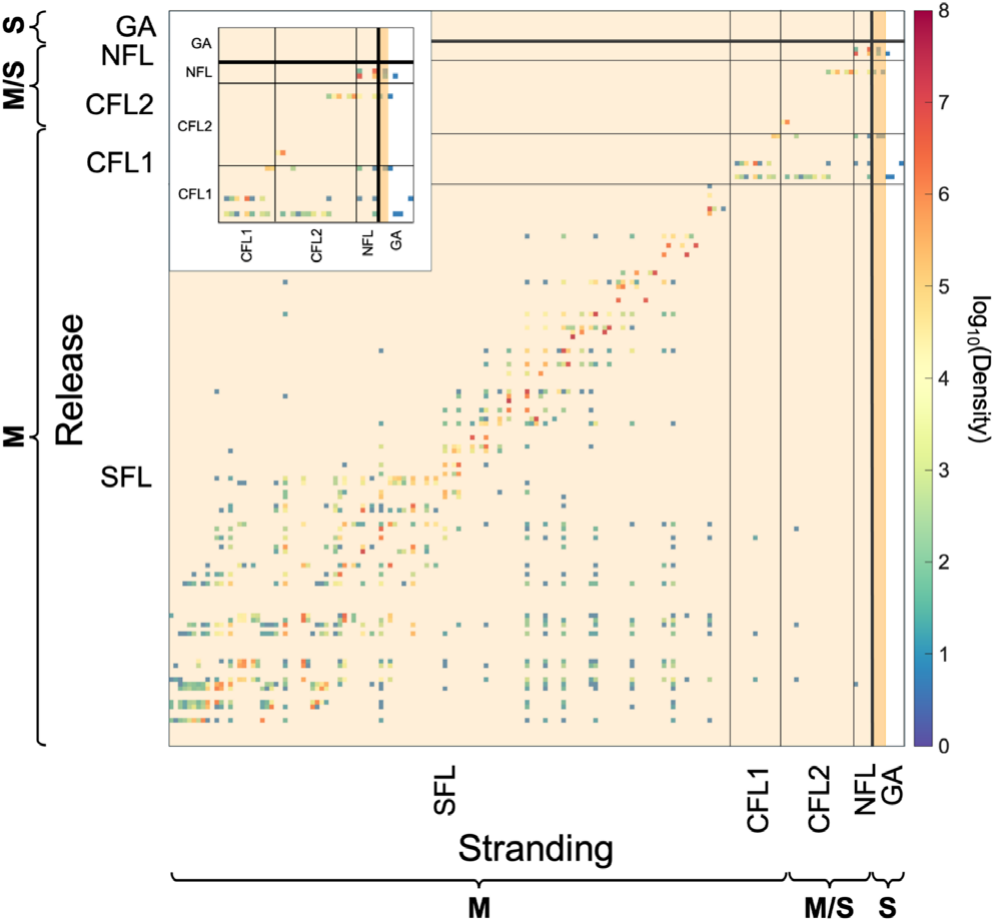
Connectivity matrix between present mangrove populations (y‐axis) and predicted stranding locations (x‐axis). Subregion codes are defined as: SFL: Southeast Florida, CFL1: Central East Florida 1, CFL2: Central East Florida 2, NFL: Northeast Florida, and GA: Georgia (Appendix S1, Figure S1 for a geographic key to subregion codes). Lighter peach shading represents latitudes suitable under present climatic conditions. Darker peach shading represents latitudes identified as suitable under SSP5‐8.5. No shading represents latitudes projected to be unsuitable based on climate. The thick horizontal black line marks the Florida‐Georgia border, which is near the current range limit of mangroves. Curly brackets indicate the transition between present‐day mangrove‐dominated (M), mangrove‐saltmarsh ecotone‐dominated (M/S), and saltmarsh‐dominated latitudes (S), as described in Cavanaugh et al. (2019). *Inset: Zoomed‐in view of the region near the current range limit, spanning CFL1 to GA*. The connectivity matrix was generated using output from a Lagrangian particle‐tracking model with a floating period of 17 months and a minimum floating period that consisted of a Monte Carlo simulation that generated random values between 1 and 5 days.

We next generate connectivity matrices to assess potential connectivity between established mangrove populations and future suitable sites along the Atlantic coast of the United States (i.e., the study area; Figure 3). Highest densities are found along the diagonal, indicating that most propagules stranded near their release site. For simulated floating periods of 3, 6, 12, and 17 months, the Gulf Stream current facilitated propagule dispersal from locations in Central East Florida (CFL2 for the 3‐month floating period; CFL1 and CFL2 for the 6, 12, and 17‐month floating period) and Northeast Florida (NFL) to locations beyond the current range limit. Longer floating times increased the stranding densities in regions further north, although overall densities beyond the current range limit remained low. In simulations with a 1‐month floating period, long‐distance connectivity was limited and most propagules stranded close to their release site. In contrast, a floating period of 3 months increased the number of propagules reaching salt marsh‐dominated locations. Despite this, present‐day conditions were predicted as unsuitable for successful establishment of the stranded propagules (Figure 3). For SSP2‐4.5, SSP3‐7.0, and SSP5‐8.5, several of these currently unsuitable wetlands were projected to become suitable (Figure 1b–d). The northernmost suitable wetlands reached by propagules were found at 30.964° N, 31.226° N, and 31.029° N, respectively. Under SSP5‐8.5, propagules with floating periods of 3, 6, 12, and 17 months reached newly suitable habitat beyond their northern range limit. Propagules with a 1‐month floating period showed no connectivity to sites projected to become suitable for mangrove establishment under future conditions. For floating periods of 6 months or longer, suitable habitat reached by propagules was projected to extend up to 31.029° N, which is 1.06° (ca. 118 km) beyond the northernmost mangrove in the FDEP dataset (29.97° N) and 0.619° (ca. 68.4 km) beyond the northernmost individual observed in the field, 30.41° N (Cavanaugh et al. 2019). Simulations with maximum floating periods of 3, 6, 12, and 17 months suggest that these locations could be colonized from multiple source populations. Propagules floating for longer than 3 months also showed stranding at higher latitudes (43.933° N), including areas where wetlands are not projected to be suitable for mangrove establishment.

### 2.4 Potential Role of Hurricanes in Range Expansion

**FIGURE 4:**
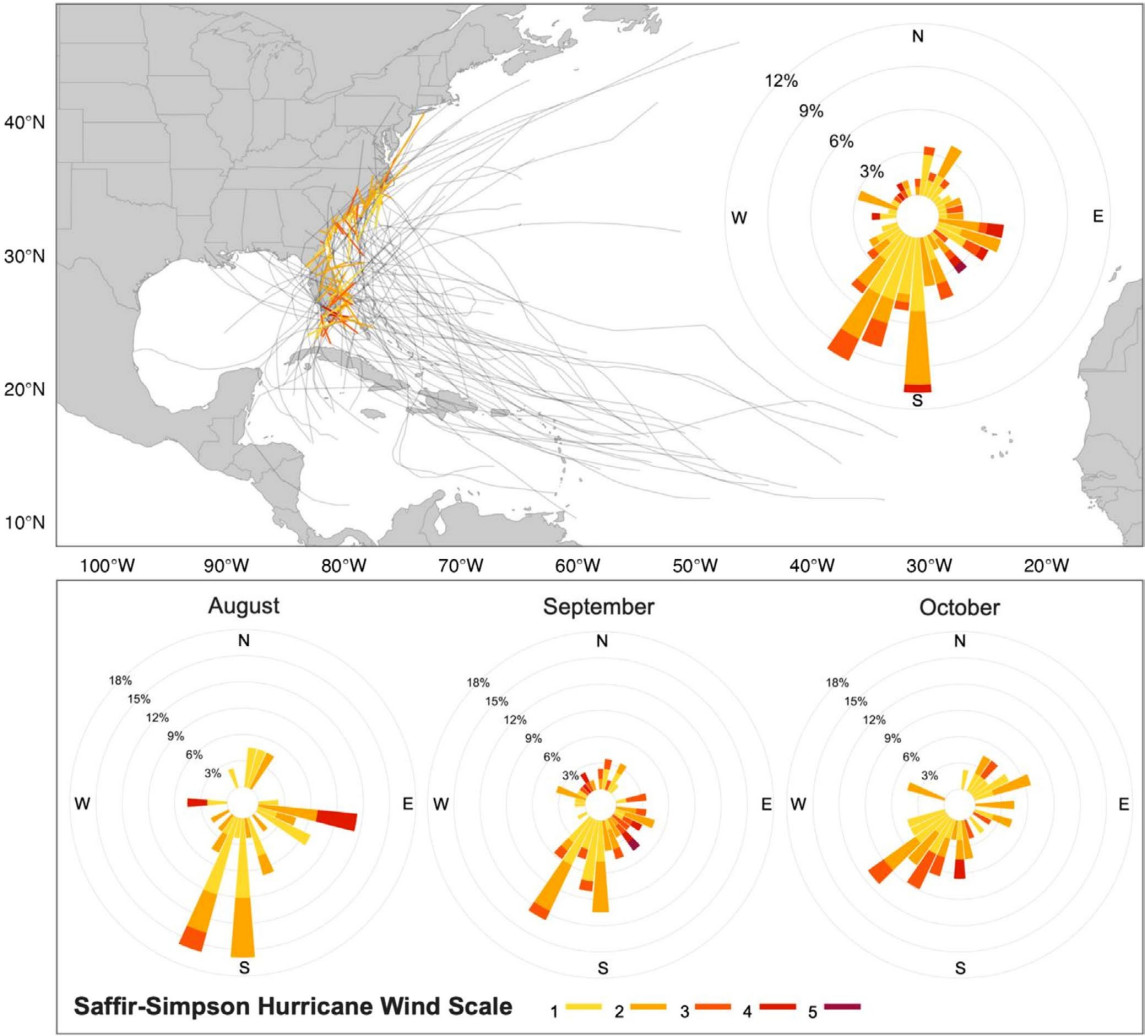
Map of historical hurricane tracks across the study region (top), alongside wind roses showing the distribution of hurricane path direction and speed during the dominant propagule‐presence period (August–October) (top) and for the individual months of that period (bottom; August to October from left to right). Colors correspond to the classification following the Saffir‐Simpson hurricane wind scale based on maximum sustained wind speed. Map lines delineate study areas and do not necessarily depict accepted national boundaries.

The wind rose diagrams of the historical hurricane tracks through the study area show a predominant path direction from the south‐southwest during the dominant period of mangrove propagule presence, i.e., August–October (Figure 4). Most storms had an intensity of Category 1 or 2 on the Saffir‐Simpson hurricane wind scale, corresponding to maximum sustained wind speeds of 119–153 km h^−1^ and 154–177 km h^−1^, respectively. Hurricanes of Category 3 or higher occurred primarily in the southern part of Florida.

## 3 Discussion

Our future scenarios analysis supports the hypothesis that warming winter temperatures will drive the continued poleward expansion of mangroves along North America's Atlantic coast. The close match between the observed mangrove distribution and the predicted distribution of our suitability model for present conditions supports previous studies showing that the mangrove range limit population in northeastern Florida is primarily climate limited (Cavanaugh et al. 2019; Bardou et al. 2021). Observations of mangrove range expansion and contraction have recurrently identified extreme freeze events as the main control on their abundance and distribution in this region (Stevens et al. 2006; Saintilan et al. 2014; Cavanaugh et al. 2014, 2019). Consequently, warming winters are projected to increase mangrove abundance, above‐ground biomass, and tree height, both within and beyond the current mangrove distribution (Bardou et al. 2024). These results are consistent with our species distribution models, predicting that ongoing climate change will create more climatically suitable mangrove habitat north of their current range limit, potentially reaching as far north as South Carolina. Recent observations by Vervaeke et al. (2025) reported, for the first time, *Avicennia germinans* and *Rhizophora mangle* individuals in Georgia (30.73° and 30.74° N, respectively). These individuals have a patchy distribution and shrubby morphology due to living near their physiological limits (Cook‐Patton et al. 2015). Their presence provides strong evidence that rising temperatures are actively driving mangrove range expansion in this region (Vervaeke et al. 2025). Although *Avicennia* is generally recognized as the more cold‐tolerant mangrove genus, the co‐occurrence of both species at the northernmost range edge may be influenced by the frequency and duration of freeze events and local microclimatic conditions. For example, warmer waters and vegetation structure can buffer temperatures at small spatial scales, allowing survival during otherwise lethal freeze events (Devaney et al. 2017; Osland et al. 2020). Over time, differential responses to freezing conditions could lead to shifts in species dominance within this region.

The simulated propagule trajectories suggest that dispersal will not be a limiting factor for mangrove range expansion and that ocean currents allow for successful colonization of these newly suitable sites from established populations. Our connectivity matrices show that under SSP2‐4.5, SSP3‐7.0, and SSP5‐8.5 climate scenarios, propagules reach wetlands beyond the current range limit that are projected to have climatically suitable conditions to sustain mangrove stands. Although overall stranding densities in these newly suitable areas are low, a single successful dispersal event can be sufficient to found a new population (Nathan et al. 2008). In addition to oceanic dispersal, propagules may also move through inland water bodies, such as estuaries and man‐made canals (e.g., the Intracoastal Waterway along the U.S. Atlantic and Gulf coasts), providing an alternative and currently unexamined dispersal pathway that can facilitate range expansion and inland migration. Interestingly, our dispersal simulations reveal several transport routes for propagules released from mangrove populations along the eastern coast of Florida. For particles with a 6‐month floating period and higher, we find evidence for propagule transport along western Florida into the Gulf. This movement may be driven by the overlap between the peak propagule release period (August–October; Gill and Tomlinson 1971; Rabinowitz 1978; Stevens et al. 2006; Goldberg and Heine 2017) and the seasonally reversing west Florida shelf current that flows northwestward during summer (June–September; Liu and Weisberg 2007). Additionally, the formation of large cyclonic eddies along the inshore side of the Loop Current, as it flows into the Straits of Florida, can result in high propagule retention near the Florida Keys or alter propagule trajectories, potentially facilitating their northwestward transport (Lee et al. 1994; Fratantoni et al. 1998). These results suggest that mangrove populations along the Atlantic coast of Florida may act as source populations for reported mangrove expansion hotspots across the Gulf coast, including coastal areas of northwest Florida, Louisiana, and Texas (Osland et al. 2022). While our simulations focus on propagules released along the Atlantic coast, transport routes along the Gulf coast suggest multiple potential propagule sources to these expansion hotspots, with likely contributions from both Atlantic and Gulf populations. Future studies that integrate population genetics with dispersal modeling could help resolve the relative contribution of different source regions and improve our understanding of the role of long‐distance dispersal in shaping the biogeography and resilience of expanding mangrove populations.

Identifying these dispersal routes highlights the relevance of using ocean models that resolve seasonal variability and fine‐scale physical processes such as tides, mesoscale eddies, and fronts to achieve realistic simulations of propagule transport and connectivity (Van der Stocken, Carroll, et al. 2019). However, the model used in this study does not account for longer‐term variability or potential climate‐driven changes in ocean circulation that could alter transport trajectories. Predicting how climate change might affect ocean currents is complex (Piecuch 2020), but recent studies suggest a potential slowdown of the Gulf Stream due to weakening of the Atlantic meridional overturning circulation (AMOC) as a result of anthropogenic climate change (Fox‐Kemper et al. 2021). The implications of such a large‐scale circulation destabilization for propagule transport in our study region are uncertain, but potential changes in ocean current strength could alter dispersal in the future.

Although our models predict an extension of climatically suitable and reachable areas in the future, the rate of mangrove colonization is difficult to predict. Positive feedback mechanisms can promote poleward expansion (Kelleway et al. 2017). At present, most climate‐limited range edge populations are small, shrub‐like individuals due to repeated disturbances and physiological stress near their tolerance limits (Cook‐Patton et al. 2015). However, observations in northern Florida near Matanzas Inlet show that northern range‐edge populations can reproduce earlier and produce larger propagules than southern populations, indicating substantial maternal investment even at the range limit (Dangremond and Feller 2016). Propagule size and early life‐history traits strongly influence seedling establishment and growth. Larger *R. mangle* propagules contain greater maternal reserves, supporting early seedling growth (Lin and Sternberg 1995), and mangrove seedlings exhibit plasticity in biomass allocation across variable habitats (Simpson et al. 2017). Together, these traits suggest that while present‐day range‐edge propagules may be less viable, northern populations may still contribute to northward expansion under favorable climatic conditions. If warming renders these currently limiting latitudes more suitable, larger and denser stands may establish (Bardou et al. 2024), increasing propagule production (Alleman and Hester 2011). This potential increase in propagule output near future range limits could enhance (long‐distance) dispersal and facilitate further expansion, particularly as newly colonized sites can themselves become source locations. Additionally, connectivity matrices show that colonization of future suitable sites can originate from both leading‐edge populations and populations farther south, highlighting how occasional long‐distance dispersal events can deliver viable propagules beyond the current range limit. At the stranding sites, salt marsh vegetation can facilitate initial colonization of mangroves by trapping propagules (Stevens et al. 2006; McKee et al. 2007) and attenuating hydrodynamic energy (Balke et al. 2011; Friess et al. 2012). Beyond these abiotic processes, biotic interactions such as interspecific competition and predation also shape expansion outcomes. For instance, herbivory can significantly reduce propagule and seedling survival, partially offsetting the positive feedbacks that promote mangrove encroachment into salt marshes (Devaney et al. 2017), and post‐establishment competition may further constrain mangrove establishment (Wei et al. 2024). However, repeated observations of salt marsh replacement by mangroves along North America's Atlantic coast suggest that mangroves tend to outcompete marsh vegetation in this region in the absence of extreme cold events (Saintilan et al. 2014; Cavanaugh et al. 2014; Vervaeke et al. 2025).

Other processes influencing the dispersal of mangrove propagules may also play an important role in controlling future range expansion. For example, hurricanes are recognized vectors for long‐distance dispersal, acting as high‐energy “pulse” events that can significantly increase the likelihood of propagule transport into more distant coastal wetland areas (Nathan et al. 2008; Van der Stocken, Wee, et al. 2019). In addition, hurricane‐driven storm surges and extreme tidal events have been reported to transport propagules inland and promote their establishment by increasing saltwater intrusion, causing mortality of less salt‐tolerant marsh species (Yannick et al. 2024). Although major hurricanes can cause extensive damage and destruction, the persistence of coastal mangrove populations along the central Atlantic hurricane path suggests that mangroves can tolerate moderate storms and recover from major storms (Krauss and Osland 2019). Within our study area, peak propagule production and release (August–October) coincide with the Atlantic hurricane season, increasing the likelihood of storm‐driven dispersal (Van der Stocken, Wee, et al. 2019). Recorded hurricane data show that storms predominantly move from the south‐southwest, suggesting high potential for along‐coast propagule transport toward more northern regions during these events. These results are supported by post‐hurricane field observations by Kennedy et al. (2020), who reported increased numbers of drift propagules at, and beyond, Florida's northeastern mangrove range limit following Hurricane Irma in 2017. In the North Atlantic basin, historical records show an apparent increase in hurricane frequency; however, gaps in early observational data and the influence of multi‐decadal natural variability limit the reliability of these long‐term trends (Murakami et al. 2020). Modeling studies suggest that while the frequency of tropical cyclones in the North Atlantic may decrease, the proportion of stronger storms (Category 3–5) will likely increase (Murakami et al. 2018, 2020), in line with global climate change predictions for tropical regions (Seneviratne et al. 2021). This could amplify directional dispersal events and further enhance the role of hurricanes in mediating range dynamics (Kennedy et al. 2020).

Our dispersal simulation covers the period from April 2011 to March 2013, during which no hurricane directly hit our study area. As a result, our simulations do not capture potential effects of interannual variability in storm frequency and intensity on long‐distance propagule transport. Future studies using long‐term ocean simulations could improve our understanding of interannual ocean variability and the effects of hurricanes on dispersal in the region. By filtering out intervals of tropical storm activity, such simulations could help distinguish the relative influence of ocean currents and episodic storm events on propagule dispersal in this region. However, the use of longer‐term simulations comes at the cost of reduced resolution and may not fully capture the effects of important coastal processes, such as tides and longshore currents, that are resolved by the numerical ocean model in our study. While the ocean circulation model (MITgcm) incorporates surface winds (Menemenlis et al. 2008), our particle‐tracking simulations do not account for potential windage effects, i.e., direct effects of wind on propagule trajectories through mechanical friction on subaerial propagule surface. However, while windage effects could strongly influence the trajectories of dispersing mangrove propagules (Van der Stocken and Menemenlis 2017), we expect these effects to be rather limited for the propagule morphotypes in this study. Flume racetrack experiments showed that small‐sized propagules, such as those found in the pioneer species *Avicennia* spp. and *Laguncularia racemosa* , are relatively insensitive to direct wind effects due to the limited subaerial surface. While direct wind effects may be larger for elongated propagules, as observed in *Rhizophora* spp., wind effects are negligible for the vertically floating propagules in this genus (Van der Stocken et al. 2015), which may be the predominant floating orientation among the population of long‐distance dispersers (Tonné et al. 2017).

Although warming winter temperatures are expected to drive the poleward expansion of mangroves in the southeastern USA, successful establishment and persistence will also depend on the interaction with other climate‐change‐related processes (Krauss et al. 2011). The intertidal position of mangrove ecosystems makes them particularly vulnerable to projected sea‐level rise. Rapidly rising water levels may lead to landward retreat of coastal wetlands or, in some cases, conversion to open water, threatening their habitat availability (Osland et al. 2022; Saintilan et al. 2023). Consequently, the persistence and expansion of mangroves will depend on the integrity of coastal habitats both within and beyond their current range, as well as their capacity to keep up with sea‐level rise through vertical accretion and/or inland migration to higher elevations (Osland et al. 2022). The ability of mangroves to cope with rising sea levels is closely linked to the severity of warming scenarios. Projections show that global temperature increases of 1.5°C–2°C will pose a risk of sea‐level rise rates exceeding the capacity of mangroves to survive through vertical accretion in this region (Saintilan et al. 2023). Additionally, opportunities for inland migration may be limited by anthropogenic development in low‐lying coastal areas with the risk of coastal squeeze (Borchert et al. 2018), especially along Florida's highly urbanized coastline.

Our results highlight the relevance of integrating climatic and dispersal factors when projecting future mangrove range dynamics. Both mangroves and salt marshes are highly valued ecosystems, and changes to their distribution could have significant ecological and socioeconomic impacts (Osland et al. 2013, 2022). Identifying future suitable and colonizable mangrove habitats presents an opportunity to guide informed conservation strategies, including site‐specific decisions on whether to allow or restrict mangrove colonization in salt marsh‐dominated wetlands. Mangrove expansion is considered beneficial due to its potential for increased carbon sequestration, nutrient storage, and enhanced protection against storm damage, flooding, and soil erosion (Doughty et al. 2016; Kelleway et al. 2017; Sánchez‐Núñez et al. 2019; Vaughn et al. 2020; Osland et al. 2022). Conversely, the loss of open salt marsh plains is a transformative ecological change that is often associated with reduced habitat availability for species that depend on these ecosystems (Krauss et al. 2011; Kelleway et al. 2017; Osland et al. 2022). Because the type and degree of ecosystem services provided by these wetlands can vary substantially across sites, comprehensive site‐specific assessments can inform management decisions (Kelleway et al. 2017). A transdisciplinary approach can be used for effectively guiding future changes in coastal wetland vegetation to support ecosystem resilience while addressing the diverse needs of local communities.

## 4 Methodology

### 4.1 Study Area

Our study area includes coastal wetlands along the Atlantic coasts of the southeastern USA, between 24° and 35° N. This latitudinal range extends approximately 5° north of the current mangrove range limit, effectively capturing the mangrove‐salt marsh ecotone as identified by Cavanaugh et al. (2019), which lies between 28.00° and 30.41° N. The three dominant mangrove species in our study region are *Avicennia germinans* , *Rhizophora mangle* , and *Laguncularia racemosa* . Among them, *A. germinans* is the most freeze‐tolerant, allowing it to extend its distribution farther north than the other species (Osland et al. 2020). Some of the salt marsh species near the mangrove‐salt marsh ecotone include *Juncus roemerianus* and *Spartina alterniflora* .

Along Florida's Atlantic coast, winter temperatures are generally mild, with occasional moderate freezes occurring a few times per decade and severe freezes occurring once every 2–3 decades (Rogers and Rohli 1991; Diettrich 1949). Florida's peninsular shape, climate, and geographic position make it particularly vulnerable to tropical hurricanes. Atlantic tropical cyclones typically form during the peak months of the Atlantic hurricane season—August, September, and October—and generally make landfall in the southeastern United States from May to December, with the highest hurricane risk in September (Goldenberg et al. 2001; Malmstadt et al. 2010).

Regional ocean circulation is characterized by the Gulf Stream, which flows through the Straits of Florida into the Atlantic Ocean, along the Florida coast. Both the east and west coasts of Florida are characterized by seasonally varying currents. The west Florida shelf circulation flows northwestward from June to September and shifts southeastward from October to May (Liu and Weisberg 2007, 2012). Along eastern Florida, waters predominantly flow northward from December to August and southward from September to November (Lee et al. 1991).

### 4.2 Mangrove and Salt Marsh Data

Mangrove occurrence data were obtained from the FFlorida Department of Environmental Protection (FDEP) 2022, which is a compilation of imagery‐based Land Use/Land Cover datasets created by 5 Water Management Districts in Florida between 2012 and 2022 (Mcowen et al. 2017). Although this dataset is based on high‐resolution aerial imagery and expert photointerpretation, it may not consistently capture small or fragmented mangrove patches, particularly near the range limit, where mangroves are typically sparse, short‐stature, and interspersed with salt marsh vegetation (Bardou et al. 2023). Hence, the FDEP data, which sets the northernmost mangrove occurrence at 29.97° N, can be considered a conservative estimate of the actual mangrove extent due to challenges associated with detection range limits. Salt marsh distribution data were obtained from the UNEP‐WCMC global salt marsh dataset v6.1 (UNEP‐WCMC 2025). This database is a synthesis of 50 salt marsh occurrence datasets using remote sensing and field‐based survey methods spanning 1973–2015 (Mcowen et al. 2017). We considered the combined extent of different mangrove and salt marsh species present in our study area as the datasets do not differentiate between species.

### 4.3 Climate Data

At the eastern North America range limit, mangrove distribution is primarily controlled by minimum air and sea surface temperature, as well as precipitation (Cavanaugh et al. 2014; Duke et al. 1998; Osland et al. 2017). For this analysis, we obtained recent (1981–2020) climate data for the extreme minimum air temperature (EMT) and mean annual precipitation (MAP) at a 1‐km spatial resolution from the North American AdaptWest database (Wang et al. 2016). Recent (2000–2020) average sea‐surface temperatures (SST) of the coldest month were provided by the Bio‐ORACLE v3.0 database at a 5 arcmin resolution (ca. 9.2 km^2^ at the equator; Assis et al. 2024; Tyberghein et al. 2012). To assess future (2071–2100) suitability for mangroves within and beyond their current range, data projections for EMT and MAP were downloaded from the AdaptWest database (Wang et al. 2016) for four Shared Socio‐Economic Pathways (SSPs): SSP1‐2.6, SSP2‐4.5, SSP3‐7.0, and SSP5‐8.5. These datasets are downscaled gridded data from the Coupled Model Intercomparison Project phase 6 (CMIP6) database corresponding to the 6th IPCC Assessment Report for future projections. We used the ensemble mean projection calculated from a subset of eight CMIP6 models as these tend to be the most representative for general‐purpose climate projections (Hausfather et al. 2022; Mahony et al. 2022). The resolution of the available datasets was insufficient to capture all occurrence points, resulting in missing climate data for some locations. Points located within inland coastal waterbodies (e.g., lagoons) missing SST data were assigned the SST value of the nearest inlet (i.e., access to the ocean). To do so, coastal waterbodies containing mangrove and/or salt marsh occurrence points were manually polygonized in QGIS (v3.36.3‐Maidenhead), and the ID of the nearest inlet was added to the attribute table. To minimize potential subjectivity and spatial uncertainty during polygon delineation, high‐resolution satellite imagery available via Google Earth (https://earth.google.com/web/) was consulted to validate the extent and boundaries of coastal waterbodies, as well as the position of their inlets. For points lacking SST data located outside of inland coastal waterbodies, as well as points missing MAP and/or EMT data, values were extracted from the nearest grid cell of the respective climate raster.

Hurricane data during 1851–2023 were obtained from the Atlantic Hurricane 2nd generation database (HURDAT2) (Landsea and Franklin 2013) maintained by the National Hurricane Center (NHC). Each hurricane record consists of the storm center's initial location, the maximum sustained wind speed, and the post‐analysis best track (six‐hourly position and intensity). Although the database goes back to 1851, observations and estimates of the best‐track parameters are more reliable for data spanning the satellites era (early 1970s). Only hurricane tracks hitting the east coast of Florida were retained for this analysis. Hurricane path direction was calculated by determining the angle relative to true north between two consecutive points on the same track and then reversing the direction by 180°. Wind roses were developed using the “openair” package in R (Carslaw and Ropkins 2012) to visualize this directional component.

### 4.4 Species Distribution Modeling

Species distribution models (SDMs) were performed using the “Biomod2” R package (Thuiller et al. 2024). Six different algorithms were generated to account for the variability from the model choice (Cavanaugh et al. 2024). These included two regression‐based approaches, generalized linear model (GLM), and generalized additive model (GAM); two classification approaches, classification tree analysis (CTA) and flexible discriminant analysis (FDA); and two machine learning approaches, boosted regression trees (GBM) and random forest (RF). To generate a presence/absence dataset, salt marsh presence points were considered as mangrove “absences.” We assume salt marsh area as potential mangrove habitat as they occupy similar intertidal niches, which is supported by the increasing evidence of mangrove encroachment into salt marsh habitats (Saintilan et al. 2014; Cavanaugh et al. 2014; Vervaeke et al. 2025). This resulted in a total of 3240 unique mangrove presence points and 13,545 unique mangrove absence points.

EMT, SST, and MAP were investigated as potential predictors for the SDMs based on regional studies that identified these variables as the primary determinants of mangrove distribution in the study region (Cavanaugh et al. 2014; Osland et al. 2017). The relationship between these variables was quantified by calculating the Spearman rank correlation coefficient. A non‐parametric test was used as the assumption of normality was violated in the predictor variables. Normality was investigated by analyzing the histogram as the large sample size (*n* = 16,785) would result in an overly sensitive Shapiro–Wilk test. Associations with Spearman R > |0.5| were considered highly correlated (Dormann et al. 2013). SST showed strong correlation with EMT (*r* = 0.965) and was excluded from the final model to reduce redundancy and avoid inflated variable importance. The predictive performance of the individual models was evaluated by randomly subsetting the dataset into a 70% training set and a 30% validation set. This evaluation method, known as cross‐validation (CV), was repeated 10 times. We quantified model performance by calculating three metrics: the area under the receiver operating characteristic curve (AUC), the true skill statistic (TSS), and overall accuracy (ACC). To minimize the uncertainty of single‐model forecasts, we combined predictions from individual models into a weighted‐average ensemble model (Araújo and New 2006). Only models with TSS threshold values above 0.8—indicating high precision—were included in the ensemble to exclude poorly performing models (Hao et al. 2019). The weighting of each prediction in the ensemble was determined by the TSS evaluation metric, giving higher weights to models with a higher TSS value (Marmion et al. 2008).

A habitat suitability threshold value was set to assess whether locations could support mangroves. Probability thresholds were calculated using the optimal thresholds function from the PresenceAbsence package in R (Freeman and Moisen 2008), based on the outcome probabilities of our SDMs. The cut‐off value was determined by comparing the mean, median, and the most conservative value of the calculated thresholds, using a range of threshold‐dependent statistics: model sensitivity, model specificity, Cohen's kappa statistic, area under the receiver operating characteristic curve (AUC), true skill statistic (TSS), and overall accuracy (ACC). The threshold with the overall highest scores was set as the suitability threshold.

### 4.5 Particle Transport Simulation

The hourly surface‐ocean current data used as input for our particle‐tracking model were obtained from a mesoscale and tide‐resolving configuration of the Massachusetts Institute of Technology general circulation model (MITgcm). The simulation was carried out in a latitude‐longitude polar cap (LLC) configuration with a polar cap that has 2160 grid cells on each face (hereafter referred to as LLC2160). The model has a nominal horizontal grid resolution of 1/24°, which ranges from 1.7 km near Antarctica to 4.6 km near the Equator, with 90 vertical levels and a vertical grid thickness of 1 m near the surface to better resolve surface currents. The simulation was initialized from a data‐constrained global ocean and sea ice solution provided by the Estimating the Circulation and Climate of the Ocean, Phase II (ECCO2) project (Menemenlis et al. 2008). The ocean simulation includes tidal forcing, allowing for an improved representation of coastal ocean dynamics. However, the high spatial and temporal resolution comes at the cost of relatively limited temporal coverage, with the model output restricted to the period spanning April 2011 to March 2013.

A Lagrangian particle‐tracking method was used to compute individual particle trajectories by linearly interpolating the LLC2160 zonal and meridional surface‐ocean velocities, which were time‐stepped using a first‐order Euler method (following Van der Stocken, Carroll, et al. 2019). Vertical motion was neglected given that mangrove propagules are buoyant and generally float at the surface. The LLC 1/24° simulation spans from April 2011 to March 2013, i.e., 28 months in total. Particles were released hourly from 87 coastal grid cells derived from mangrove presence data within our study area, between August and October 2011, coinciding with the peak propagule release period in this region (Gill and Tomlinson 1971; Rabinowitz 1978; Stevens et al. 2006; Goldberg and Heine 2017). Points located behind dispersal barriers were reassigned to the nearest inlet, and any resulting duplicate locations were removed. All simulated propagules were treated equally, regardless of species, and were allowed maximum floating periods of 1, 3, 6, 12, and 17 months, which are within the range of reported floating periods for *R. mangle* and *A. germinans* (Lonard et al. 2016; Alleman and Hester 2011; Rabinowitz 1978). While a 17‐month floating period might overestimate dispersal times, it represents the upper limit of the simulation duration and accounts for the rare long‐distance dispersal events that drive large‐scale processes, such as colonization of new habitat (Nathan et al. 2008). Additionally, this simulation accounts for the current lack of comprehensive empirical data on *maximum* propagule floating durations, which is largely due to the extended observation periods required—often exceeding the length of typical experimental studies—and anticipates refinements as future research provides more accurate estimates. Dispersal trajectory density maps were generated for the five different maximum floating periods by aggregating all Lagrangian particle trajectories on a 1/24° grid (i.e., the native grid resolution).

Potential connectivity was estimated by tracking all the particles that stranded within the respective floating period. Propagules were considered stranded when they first reached an ocean cell adjacent to a land grid cell. We included a minimum floating period to avoid immediate stranding near the release location, allowing for potential long‐distance dispersal events. Since the value of this parameter is arbitrary, we used a Monte Carlo simulation to generate random minimum floating period values between 1 and 5 days. This range of minimum floating periods represents a timescale sufficient for propagules to disperse beyond the local system and embark on long‐distance dispersal events (Van der Stocken, Wee, et al. 2019). Connectivity matrices were generated to evaluate potential stranding in the study region after dispersal from a release location. Particles that stranded on urban beaches were excluded, ensuring that only those directly reaching mangroves, salt marshes, or inlets connected to these habitats were included. The habitat suitability threshold calculated based on the outcome probabilities of our SDMs was used to assess whether stranding locations could support mangrove propagule establishment.

## Author Contributions

**Lucia I. A. Enes Gramoso:** conceptualization (equal); writing – original draft (equal); writing – review and editing (equal); formal analysis (equal). **Dustin Carroll:** conceptualization (equal); writing – review and editing (equal); formal analysis (equal). **Kyle C. Cavanaugh:** conceptualization (equal); writing – review and editing (equal). **Remi Bardou:** conceptualization (equal); writing – review and editing (equal). **Michael J. Osland:** conceptualization (equal); writing – review and editing (equal). **Tom Van der Stocken:** conceptualization (equal); writing – original draft (equal); writing – review and editing (equal); formal analysis (equal).

## Conflicts of Interest

The authors declare no conflicts of interest.

## Supporting information


**Appendix S1:** gcb70676‐sup‐0001‐AppendixS1.pdf.

## Data Availability

The data that support the findings of this study are available in Zenodo at https://doi.org/10.5281/zenodo.16950603. These data were derived from the following resources available in the public domain: Bio‐Oracle, https://bio‐oracle.org/—AdaptWest, https://adaptwest.databasin.org/pages/adaptwest‐climatena/—ECCO, https://data.nas.nasa.gov/ecco/—FDEP: Statewide Land‐use Land‐cover, https://geodata.dep.state.fl.us/datasets/FDEP::statewide‐land‐use‐land‐cover/about‐Ocean+Habitats, https://data.unep‐wcmc.org/datasets/43—HURDAT2, https://www.nhc.noaa.gov/data/#hurdat.
