# Supplementary material for: 21st‐Century Mangrove Expansion Along the Southeastern United States
Source: Glob Chang Biol. 2026 Jan 7;32(1):e70676. doi: 10.1111/gcb.70676 (PMC12776246; doi:10.1111/gcb.70676)
Supplement: Supplementary file 1 — Appendix S1: gcb70676‐sup‐0001‐AppendixS1.pdf. [file GCB-32-e70676-s001.pdf]

Supporting Information for

21st-century mangrove expansion along the southeastern United States

Lucia I. A. Enes Gramoso[1*], Dustin Carroll[2,3], Kyle C. Cavanaugh[4], Remi Bardou[5], Michael J. Osland[6], Tom Van der Stocken[1,3]

[1]Department of Biology, bDIV: Ecology, Evolution & Genetics, Vrije Universiteit Brussel, Pleinlaan 2, 1050 Brussels, Belgium; [2]Moss Landing Marine Laboratories, San José State University, Moss Landing, CA 95039; [3]Earth Science Section, Jet Propulsion Laboratory, California Institute of Technology, Pasadena, CA 91109; [4]Department of Geography, University of California, Los Angeles, CA 90095; [5]Institute for Global Change Biology, University of Michigan, Ann Arbor, MI 48109; [6]U.S. Geological Survey, Lafayette, LA 70506; [*]To whom correspondence may be addressed. Email: Lucia.Idalina.A.Enes.Gramoso@vub.be

This PDF file includes:

Figures S1 to S26

SI References

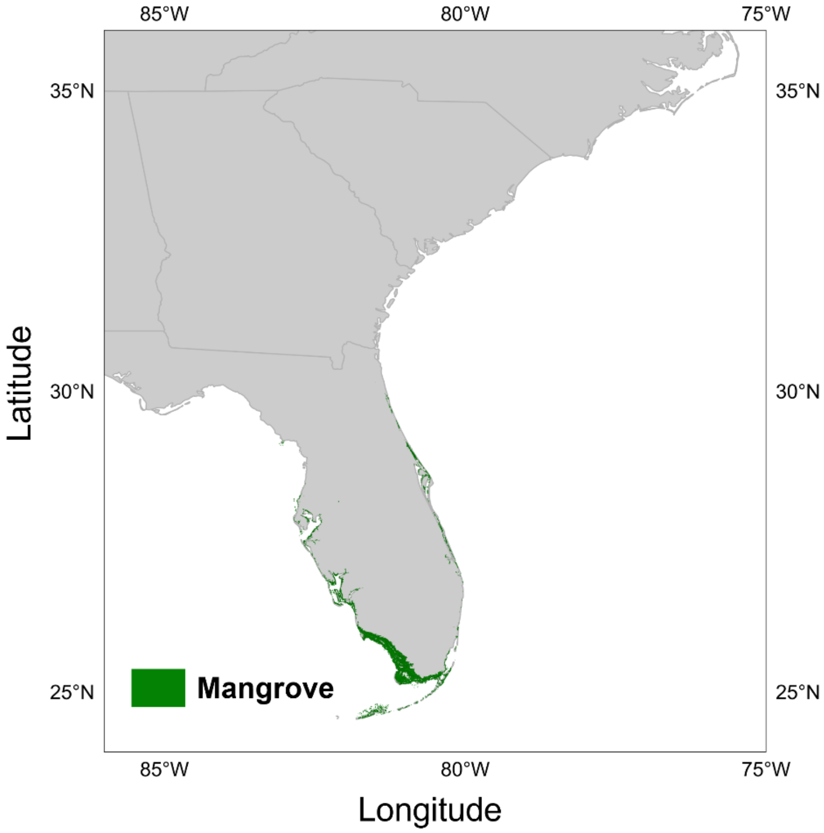

Fig. S1. Raw mangrove occurrence data obtained from the Florida Department of Environmental Protection (FDEP, 2022). Map lines delineate study areas and do not necessarily depict accepted national boundaries.

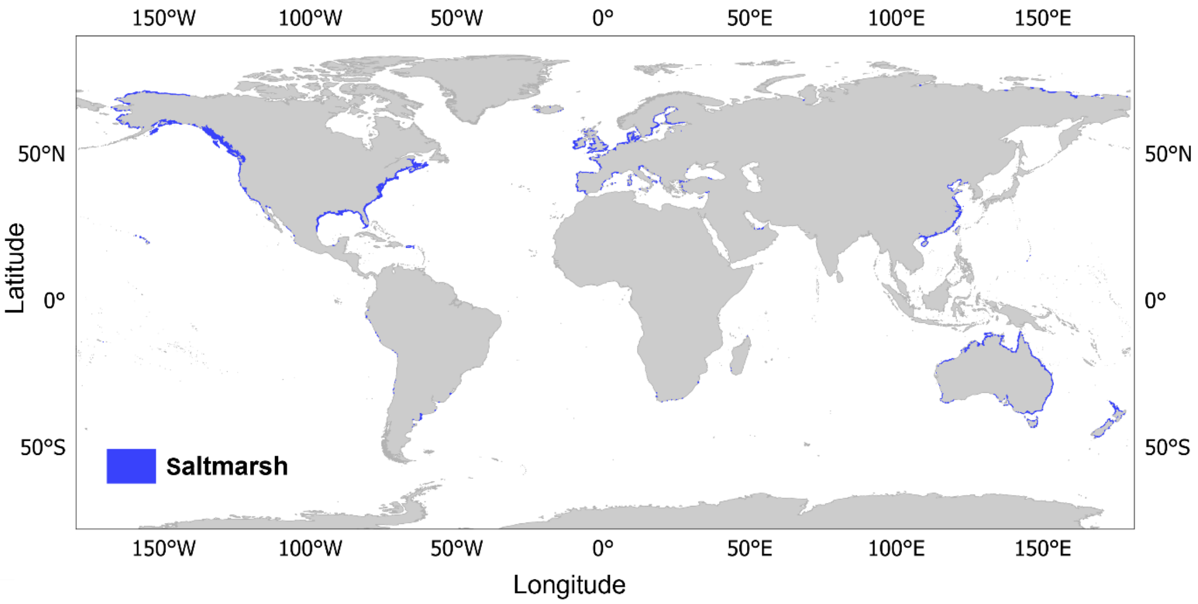

Fig. S2. Raw salt marsh occurrence data obtained from the UNEP-WCMC global salt marsh dataset v6.1 (UNEP-WCMC, 2025). Map lines delineate study areas and do not necessarily depict accepted national boundaries.

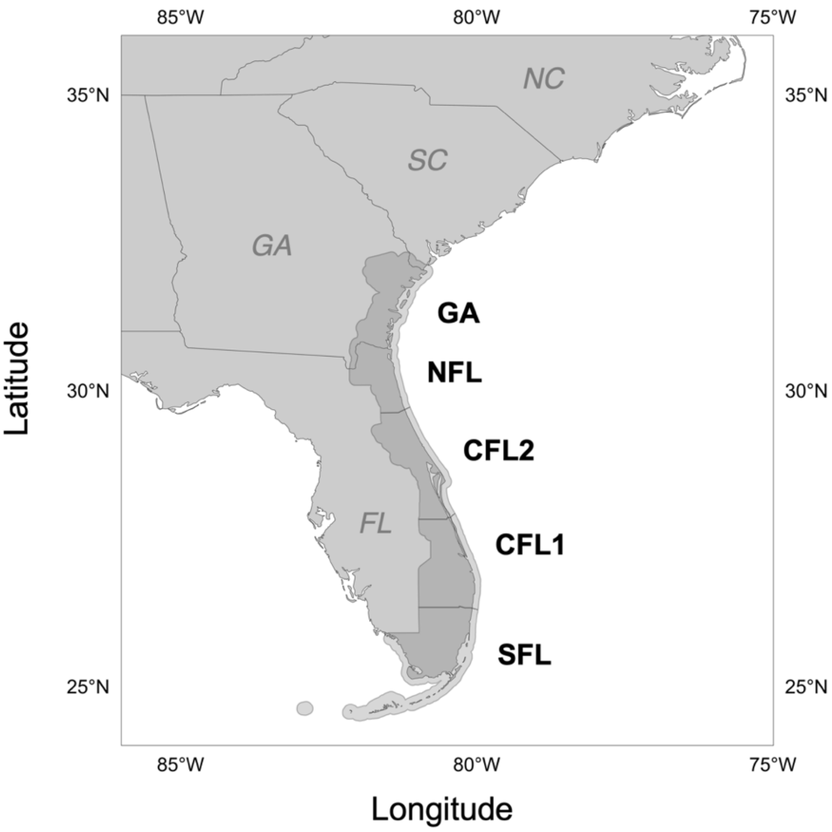

Fig. S3. Key to subregion codes, defined as: **SFL**: Southeast Florida, **CFL1**: Central East Florida 1, **CFL2**: Central East Florida 2, **NFL**: Northeast Florida, **GA**: Georgia, **FL**: Florida, **SC**: South Carolina, and **NC**: North Carolina. Map lines delineate study areas and do not necessarily depict accepted national boundaries.

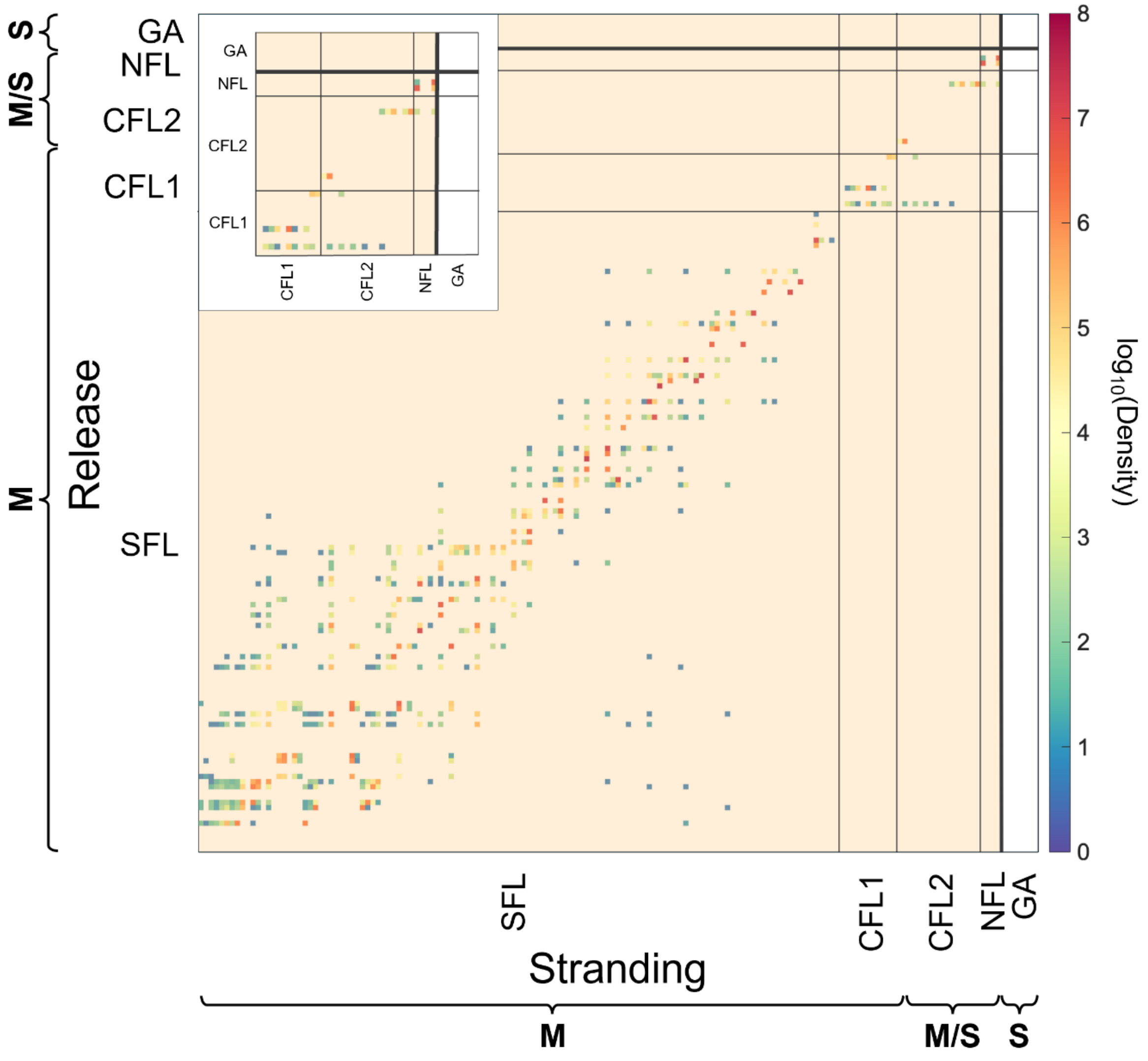

Fig. S4. Connectivity matrix between present mangrove populations (y-axis) and predicted stranding locations (x-axis). Subregion codes are defined as: **SFL**: Southeast Florida, **CFL1**: Central East Florida 1, **CFL2**: Central East Florida 2, **NFL**: Northeast Florida, and **GA**: Georgia (SI appendix, Fig. S3 for a geographic key to subregion codes). Lighter peach shading represents latitudes suitable under present climatic conditions. Darker peach shading represents latitudes identified as suitable under SSP1-2.6. No shading represents latitudes projected to be unsuitable based on climate. The thick horizontal black line marks the Florida-Georgia border, which is near the current range limit of mangroves. Curly brackets indicate the transition between present-day mangrove-dominated (**M**),

mangrove-saltmarsh ecotone-dominated (**M/S**), and saltmarsh-dominated latitudes (**S**), as described in Cavanaugh *et al*. (2019). Inset: zoomed-in view of the region near the current range limit, spanning CFL1 to GA. The connectivity matrix was generated using output from a Lagrangian particle-tracking model with a floating period of 1 month and a minimum floating period that consisted of a Monte Carlo simulation that generated random values between 1–5 days.

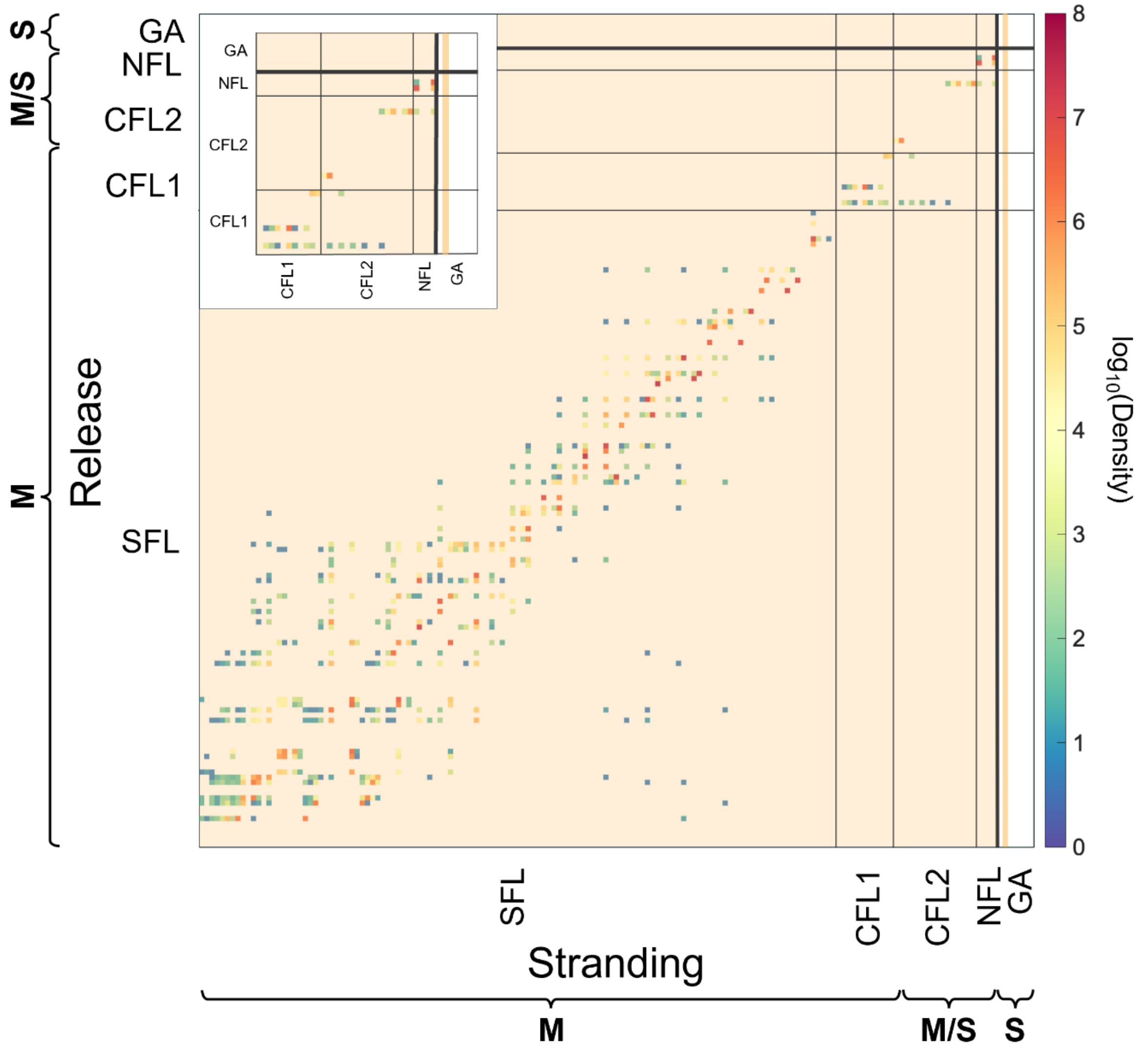

Fig. S5. Connectivity matrix between present mangrove populations (y-axis) and predicted stranding locations (x-axis). Subregion codes are defined as: **SFL**: Southeast Florida, **CFL1**: Central East Florida 1, **CFL2**: Central East Florida 2, **NFL**: Northeast Florida, and **GA**: Georgia (SI appendix, Fig. S3 for a geographic key to subregion codes). Lighter peach shading represents latitudes suitable under present climatic conditions. Darker peach shading represents latitudes identified as suitable under SSP2-4.5. No shading represents latitudes projected to be unsuitable based on climate. The thick horizontal black line marks the Florida-Georgia border, which is near the current range limit of mangroves. Curly brackets indicate the transition between present-day mangrove-dominated (**M**),

mangrove-saltmarsh ecotone-dominated (**M/S**), and saltmarsh-dominated latitudes (**S**), as described in Cavanaugh *et al*. (2019). Inset: zoomed-in view of the region near the current range limit, spanning CFL1 to GA. The connectivity matrix was generated using output from a Lagrangian particle-tracking model with a floating period of 1 month and a minimum floating period that consisted of a Monte Carlo simulation that generated random values between 1–5 days.

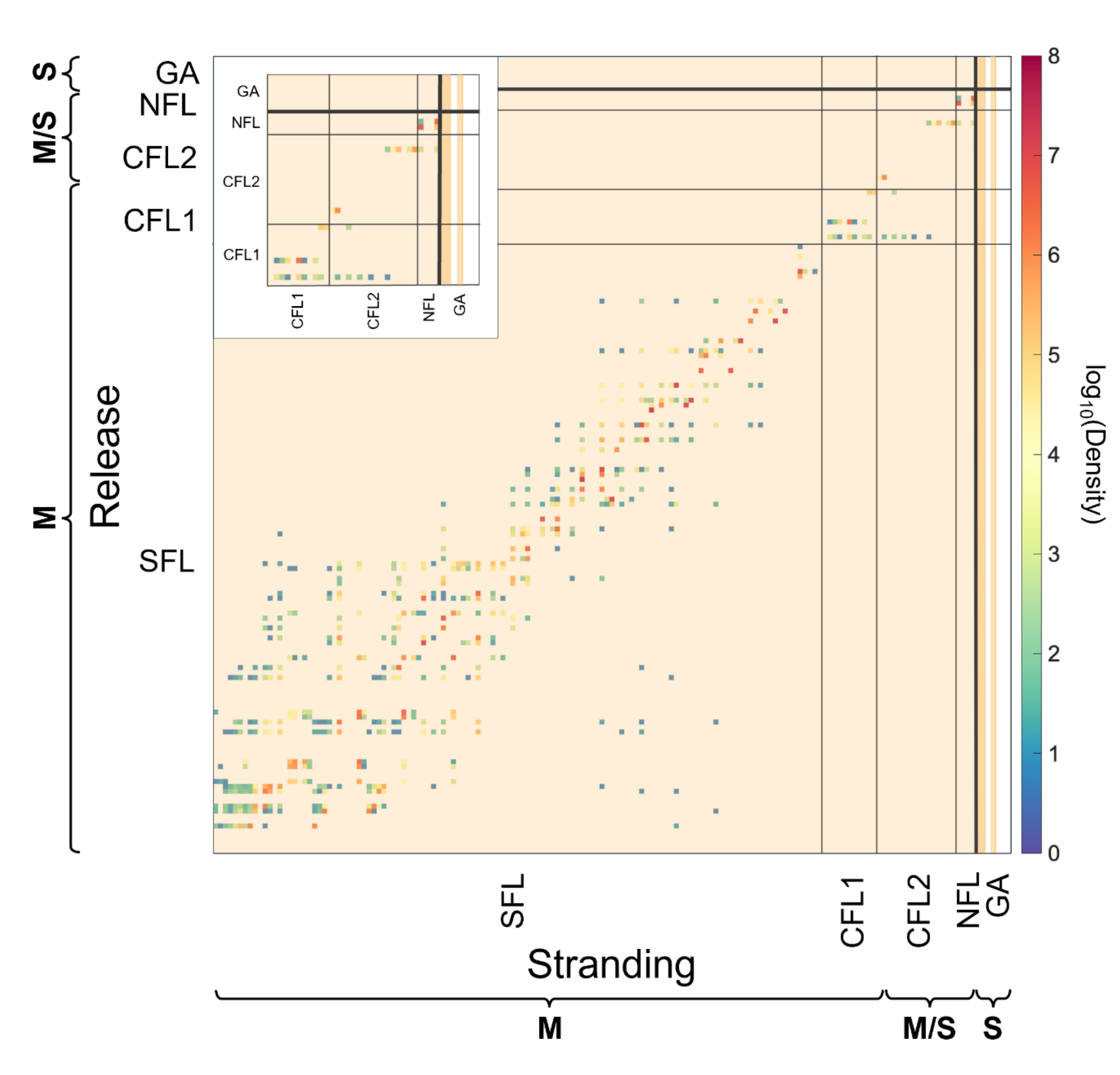

Fig. S6. Connectivity matrix between present mangrove populations (y-axis) and predicted stranding locations (x-axis). Subregion codes are defined as: **SFL**: Southeast Florida, **CFL1**: Central East Florida 1, **CFL2**: Central East Florida 2, **NFL**: Northeast Florida, and **GA**: Georgia (SI appendix, Fig. S3 for a geographic key to subregion codes). Lighter peach shading represents latitudes suitable under present climatic conditions. Darker peach shading represents latitudes identified as suitable under SSP3-7.0. No shading represents latitudes projected to be unsuitable based on climate. The thick horizontal black line marks the Florida-Georgia border, which is near the current range limit of mangroves. Curly brackets indicate the transition between present-day mangrove-dominated (**M**), mangrove-saltmarsh ecotone-dominated (**M/S**), and saltmarsh-dominated latitudes (**S**), as described

in Cavanaugh *et al*. (2019). Inset: zoomed-in view of the region near the current range limit, spanning CFL1 to GA. The connectivity matrix was generated using output from a Lagrangian particle-tracking model with a floating period of 1 month and a minimum floating period that consisted of a Monte Carlo simulation that generated random values between 1–5 days.

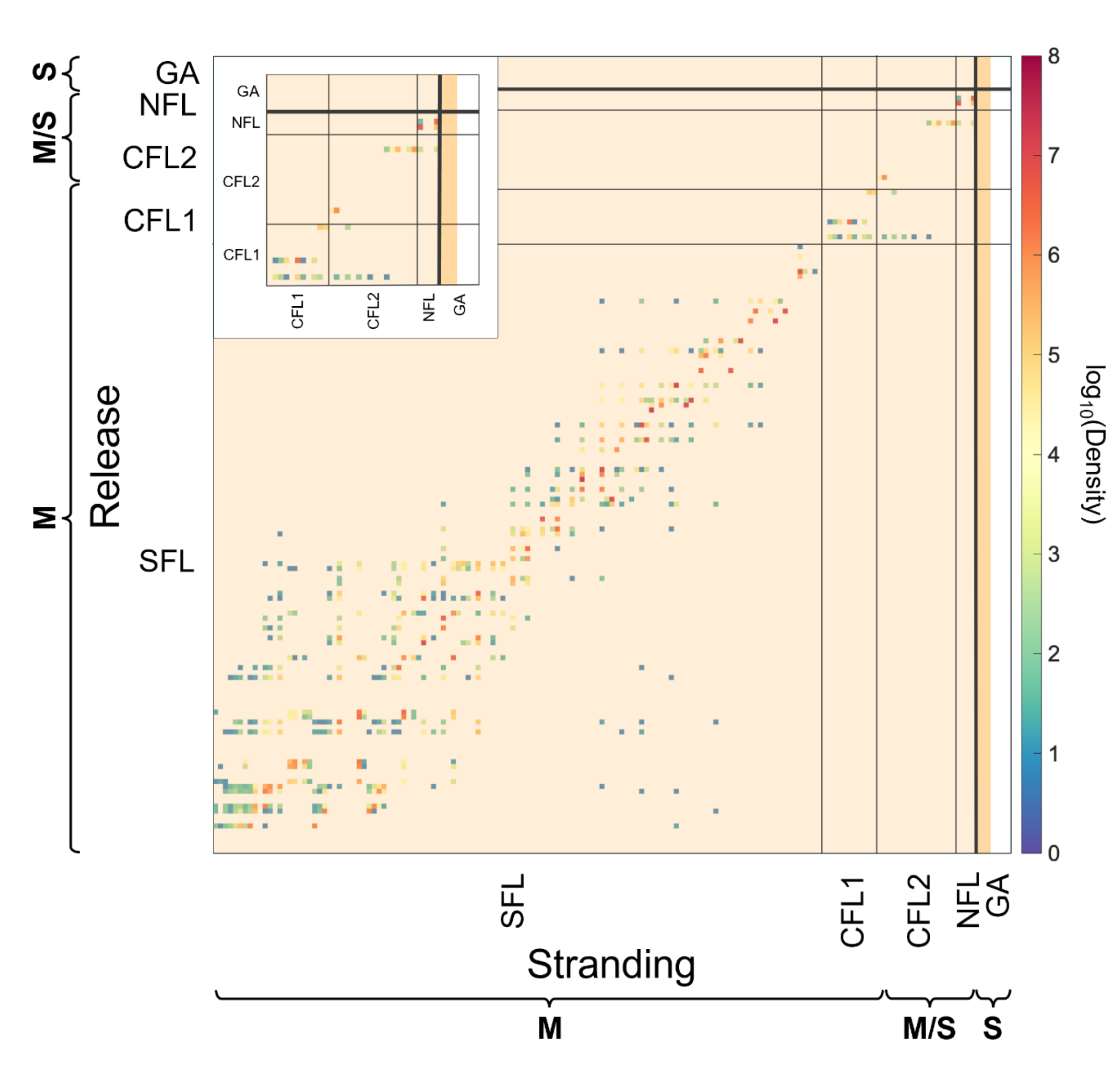

Fig. S7. Connectivity matrix between present mangrove populations (y-axis) and predicted stranding locations (x-axis). Subregion codes are defined as: **SFL**: Southeast Florida, **CFL1**: Central East Florida 1, **CFL2**: Central East Florida 2, **NFL**: Northeast Florida, and **GA**: Georgia (SI appendix, Fig. S3 for a geographic key to subregion codes). Lighter peach shading represents latitudes suitable under present climatic conditions. Darker peach shading represents latitudes identified as suitable under SSP5-8.5. No shading represents latitudes projected to be unsuitable based on climate. The thick horizontal black line marks the Florida-Georgia border, which is near the current range limit of mangroves. Curly brackets indicate the transition between present-day mangrove-dominated (**M**),

mangrove-saltmarsh ecotone-dominated (**M/S**), and saltmarsh-dominated latitudes (**S**), as described in Cavanaugh *et al*. (2019). Inset: zoomed-in view of the region near the current range limit, spanning CFL1 to GA. The connectivity matrix was generated using output from a Lagrangian particle-tracking model with a floating period of 1 month and a minimum floating period that consisted of a Monte Carlo simulation that generated random values between 1–5 days.

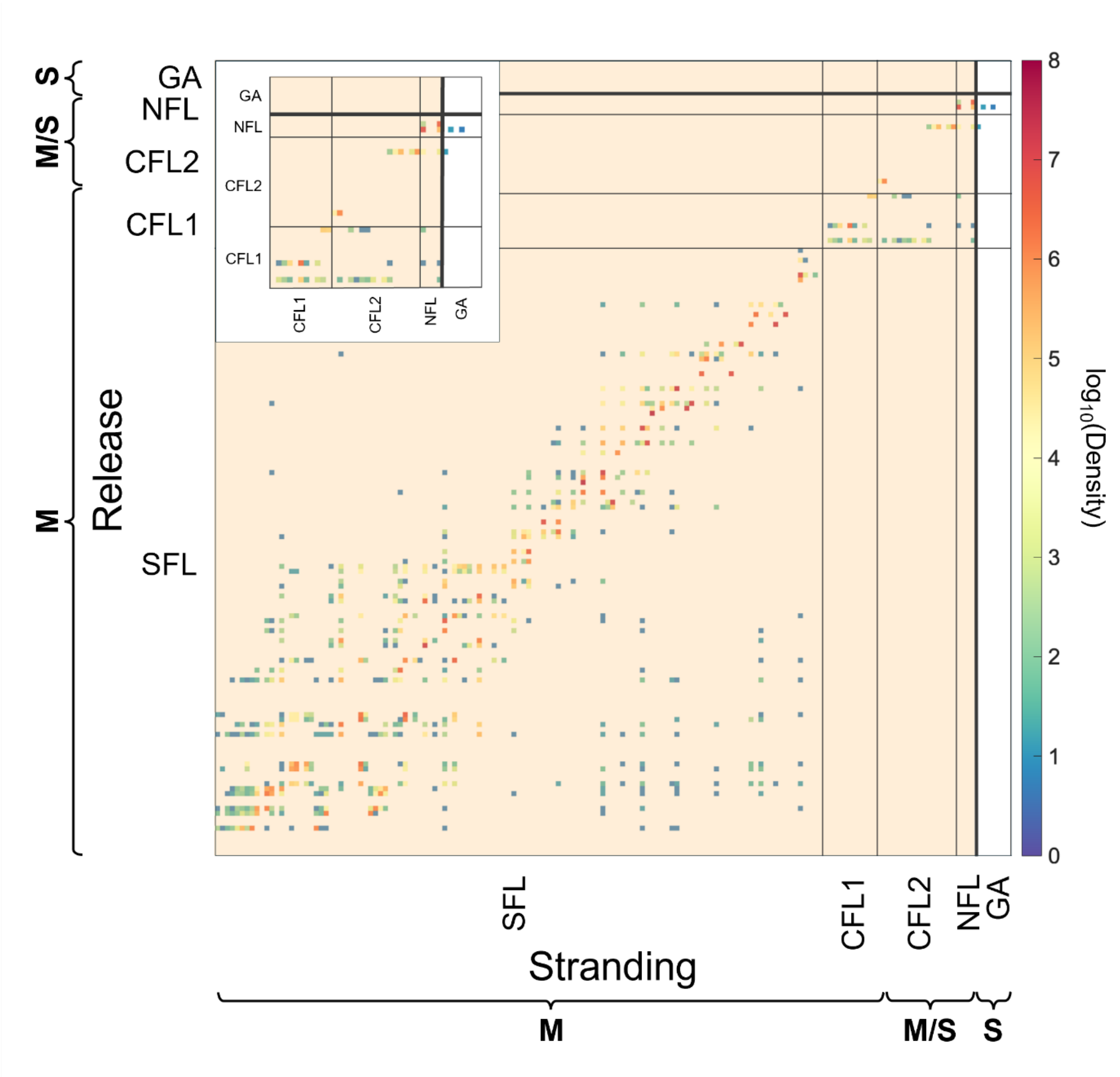

Fig. S8. Connectivity matrix between present mangrove populations (y-axis) and predicted stranding locations (x-axis). Subregion codes are defined as: **SFL**: Southeast Florida, **CFL1**: Central East Florida 1, **CFL2**: Central East Florida 2, **NFL**: Northeast Florida, and **GA**: Georgia (SI appendix, Fig. S3 for a geographic key to subregion codes). Lighter peach shading represents latitudes suitable under present climatic conditions. Darker peach shading represents latitudes identified as suitable under SSP1-2.6. No shading represents latitudes projected to be unsuitable based on climate. The thick horizontal black line marks the Florida-Georgia border, which is near the current range limit of mangroves. Curly brackets indicate the transition between present-day mangrove-dominated (**M**), mangrove-saltmarsh ecotone-dominated (**M/S**), and saltmarsh-dominated latitudes (**S**), as described

in Cavanaugh *et al*. (2019). Inset: zoomed-in view of the region near the current range limit, spanning CFL1 to GA. The connectivity matrix was generated using output from a Lagrangian particle-tracking model with a floating period of 3 months and a minimum floating period that consisted of a Monte Carlo simulation that generated random values between 1–5 days.

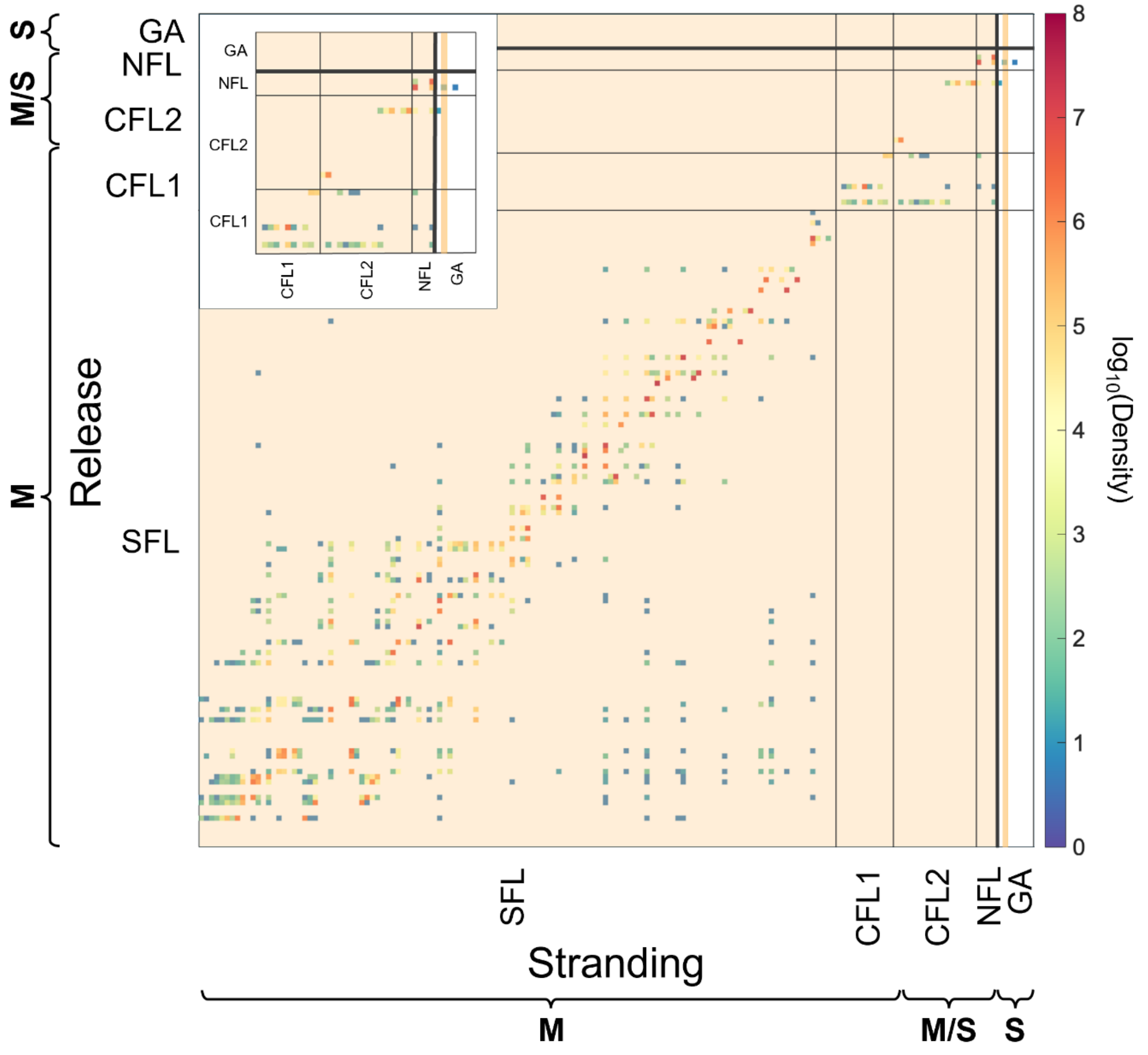

Fig. S9. Connectivity matrix between present mangrove populations (y-axis) and predicted stranding locations (x-axis). Subregion codes are defined as: **SFL**: Southeast Florida, **CFL1**: Central East Florida 1, **CFL2**: Central East Florida 2, **NFL**: Northeast Florida, and **GA**: Georgia (SI appendix, Fig. S3 for a geographic key to subregion codes). Lighter peach shading represents latitudes suitable under present climatic conditions. Darker peach shading represents latitudes identified as suitable under SSP2-4.5. No shading represents latitudes projected to be unsuitable based on climate. The thick horizontal black line marks the Florida-Georgia border, which is near the current range limit of mangroves. Curly brackets indicate the transition between present-day mangrove-dominated (**M**),

mangrove-saltmarsh ecotone-dominated (**M/S**), and saltmarsh-dominated latitudes (**S**), as described in Cavanaugh *et al*. (2019). Inset: zoomed-in view of the region near the current range limit, spanning CFL1 to GA. The connectivity matrix was generated using output from a Lagrangian particle-tracking model with a floating period of 3 months and a minimum floating period that consisted of a Monte Carlo simulation that generated random values between 1–5 days.

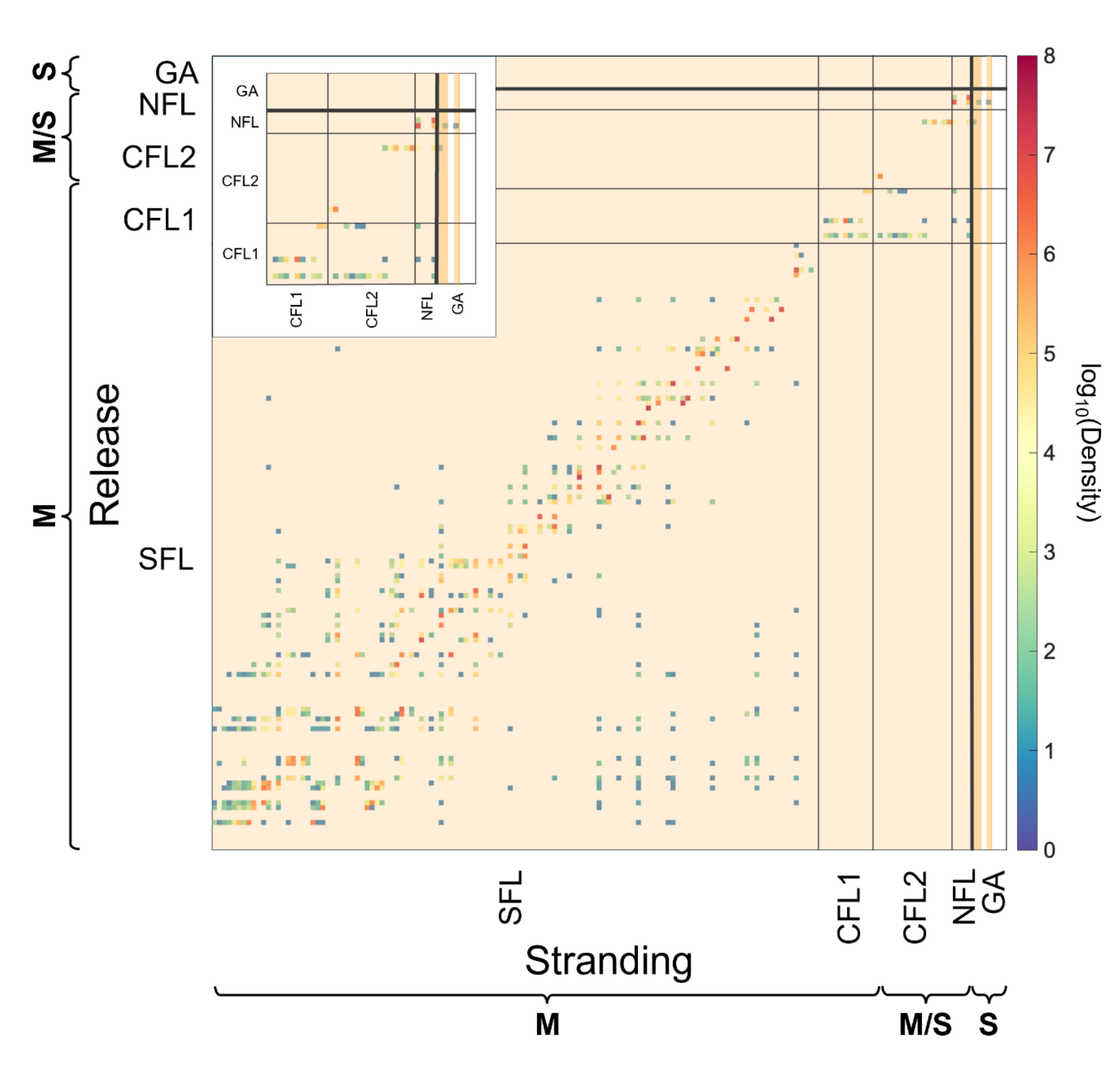

Fig. S10. Connectivity matrix between present mangrove populations (y-axis) and predicted stranding locations (x-axis). Subregion codes are defined as: **SFL**: Southeast Florida, **CFL1**: Central East Florida 1, **CFL2**: Central East Florida 2, **NFL**: Northeast Florida, and **GA**: Georgia (SI appendix, Fig. S3 for a geographic key to subregion codes). Lighter peach shading represents latitudes suitable under present climatic conditions. Darker peach shading represents latitudes identified as suitable under SSP3-7.0. No shading represents latitudes projected to be unsuitable based on climate. The thick horizontal black line marks the Florida-Georgia border, which is near the current range limit of mangroves. Curly brackets indicate the transition between present-day mangrove-dominated (**M**),

mangrove-saltmarsh ecotone-dominated (**M/S**), and saltmarsh-dominated latitudes (**S**), as described in Cavanaugh *et al*. (2019). Inset: zoomed-in view of the region near the current range limit, spanning CFL1 to GA. The connectivity matrix was generated using output from a Lagrangian particle-tracking model with a floating period of 3 months and a minimum floating period that consisted of a Monte Carlo simulation that generated random values between 1–5 days.

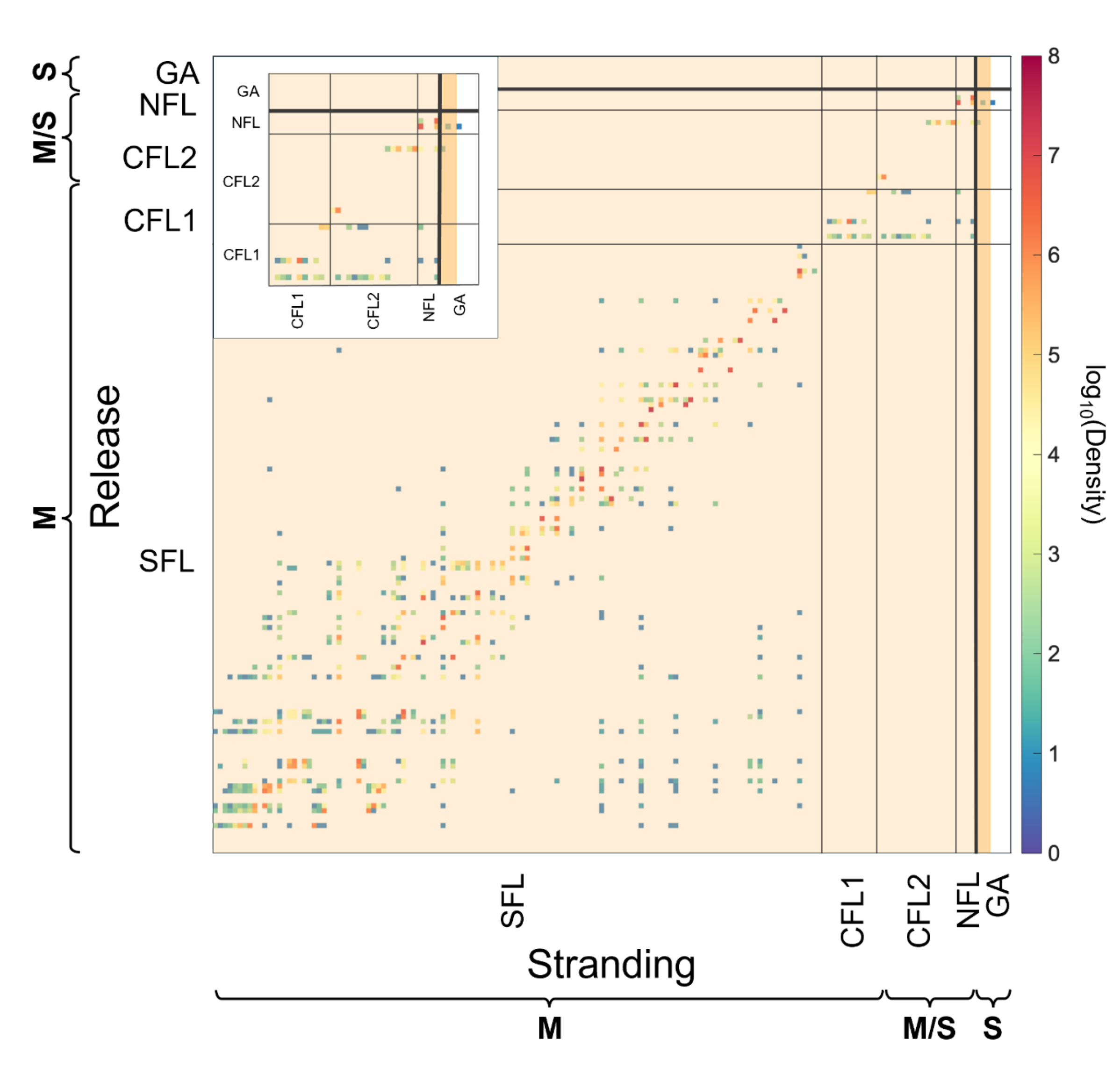

Fig. S11. Connectivity matrix between present mangrove populations (y-axis) and predicted stranding locations (x-axis). Subregion codes are defined as: **SFL**: Southeast Florida, **CFL1**: Central East Florida 1, **CFL2**: Central East Florida 2, **NFL**: Northeast Florida, and **GA**: Georgia (SI appendix, Fig. S3 for a geographic key to subregion codes). Lighter peach shading represents latitudes suitable under present climatic conditions. Darker peach shading represents latitudes identified as suitable under SSP5-8.5. No shading represents latitudes projected to be unsuitable based on climate. The thick horizontal black line marks the Florida-Georgia border, which is near the current range limit of mangroves. Curly brackets indicate the transition between present-day mangrove-dominated (**M**),

mangrove-saltmarsh ecotone-dominated (**M/S**), and saltmarsh-dominated latitudes (**S**), as described in Cavanaugh *et al*. (2019). Inset: zoomed-in view of the region near the current range limit, spanning CFL1 to GA. The connectivity matrix was generated using output from a Lagrangian particle-tracking model with a floating period of 3 months and a minimum floating period that consisted of a Monte Carlo simulation that generated random values between 1–5 days.

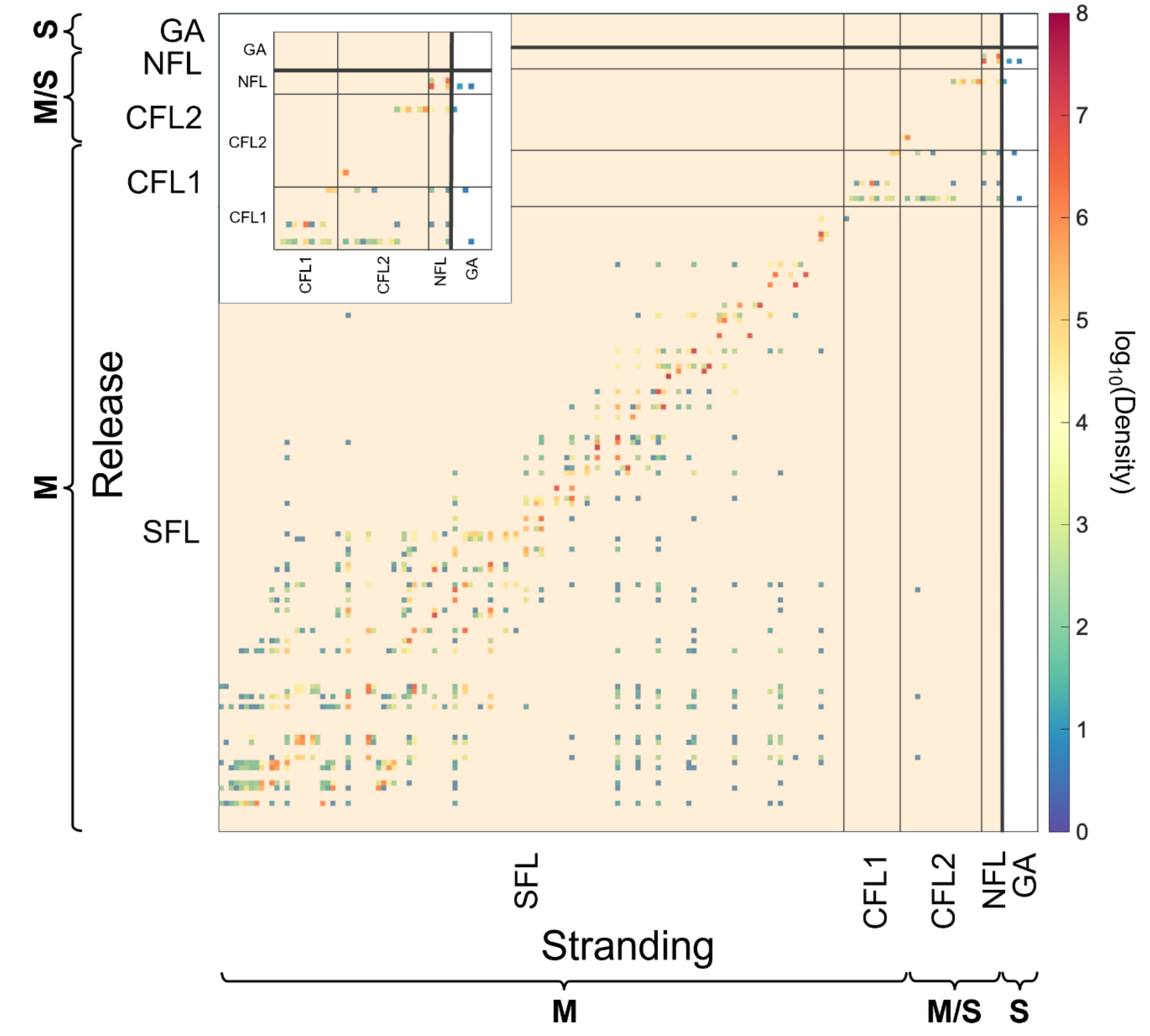

Fig. S12. Connectivity matrix between present mangrove populations (y-axis) and predicted stranding locations (x-axis). Subregion codes are defined as: **SFL**: Southeast Florida, **CFL1**: Central East Florida 1, **CFL2**: Central East Florida 2, **NFL**: Northeast Florida, and **GA**: Georgia (SI appendix, Fig. S3 for a geographic key to subregion codes). Lighter peach shading represents latitudes suitable under present climatic conditions. Darker peach shading represents latitudes identified as suitable under SSP1-2.6. No shading represents latitudes projected to be unsuitable based on climate. The thick horizontal black line marks the Florida-Georgia border, which is near the current range limit of mangroves. Curly brackets indicate the transition between present-day mangrove-dominated (**M**), mangrove-saltmarsh ecotone-dominated (**M/S**), and saltmarsh-dominated latitudes (**S**), as described

in Cavanaugh *et al*. (2019). Inset: zoomed-in view of the region near the current range limit, spanning CFL1 to GA. The connectivity matrix was generated using output from a Lagrangian particle-tracking model with a floating period of 6 months and a minimum floating period that consisted of a Monte Carlo simulation that generated random values between 1–5 days.

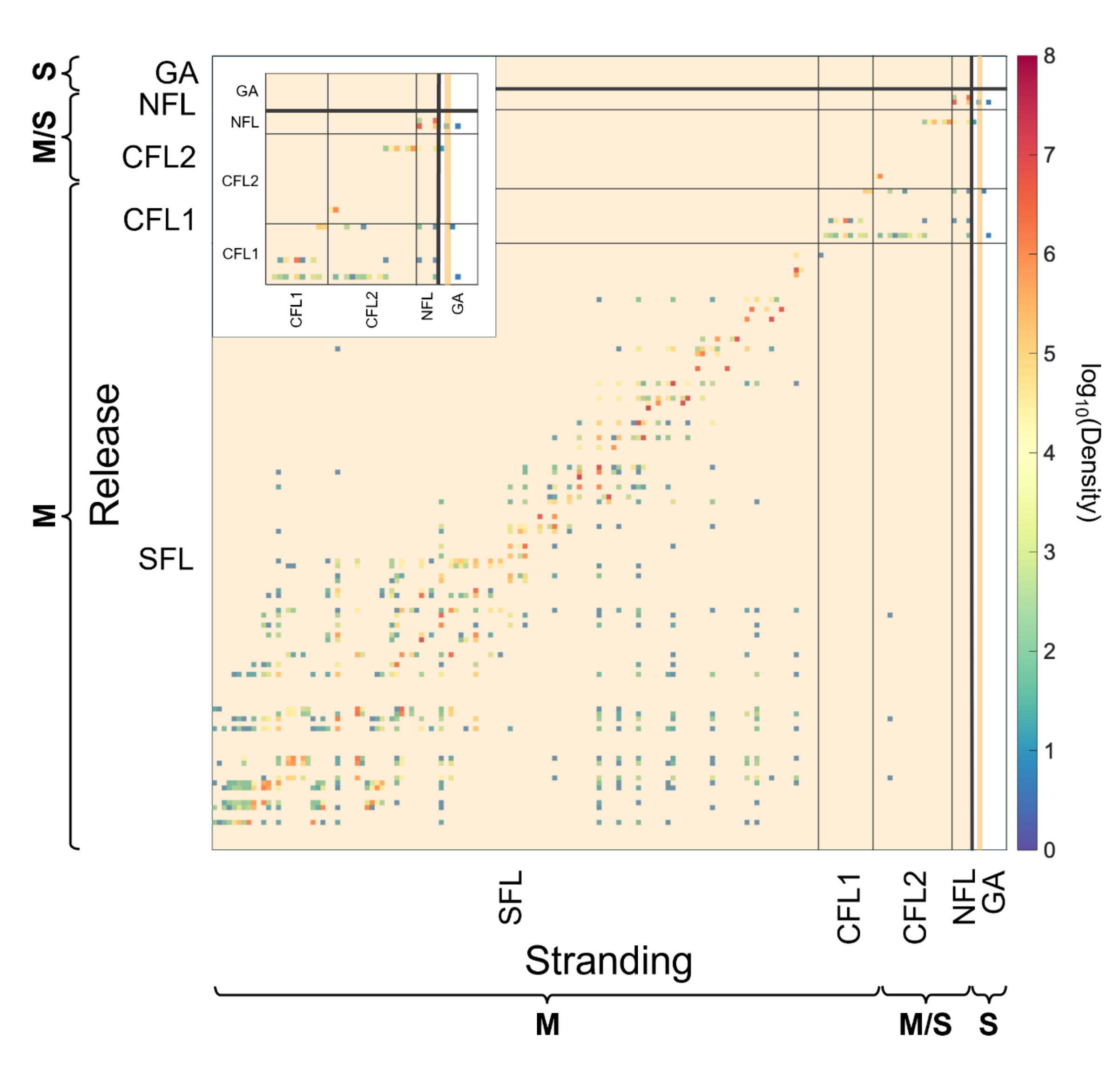

Fig. S13. Connectivity matrix between present mangrove populations (y-axis) and predicted stranding locations (x-axis). Subregion codes are defined as: **SFL**: Southeast Florida, **CFL1**: Central East Florida 1, **CFL2**: Central East Florida 2, **NFL**: Northeast Florida, and **GA**: Georgia (SI appendix, Fig. S3 for a geographic key to subregion codes). Lighter peach shading represents latitudes suitable under present climatic conditions. Darker peach shading represents latitudes identified as suitable under SSP2-4.5. No shading represents latitudes projected to be unsuitable based on climate. The thick horizontal black line marks the Florida-Georgia border, which is near the current range limit of mangroves. Curly brackets indicate the transition between present-day mangrove-dominated (**M**),

mangrove-saltmarsh ecotone-dominated (**M/S**), and saltmarsh-dominated latitudes (**S**), as described in Cavanaugh *et al*. (2019). Inset: zoomed-in view of the region near the current range limit, spanning CFL1 to GA. The connectivity matrix was generated using output from a Lagrangian particle-tracking model with a floating period of 6 months and a minimum floating period that consisted of a Monte Carlo simulation that generated random values between 1–5 days.

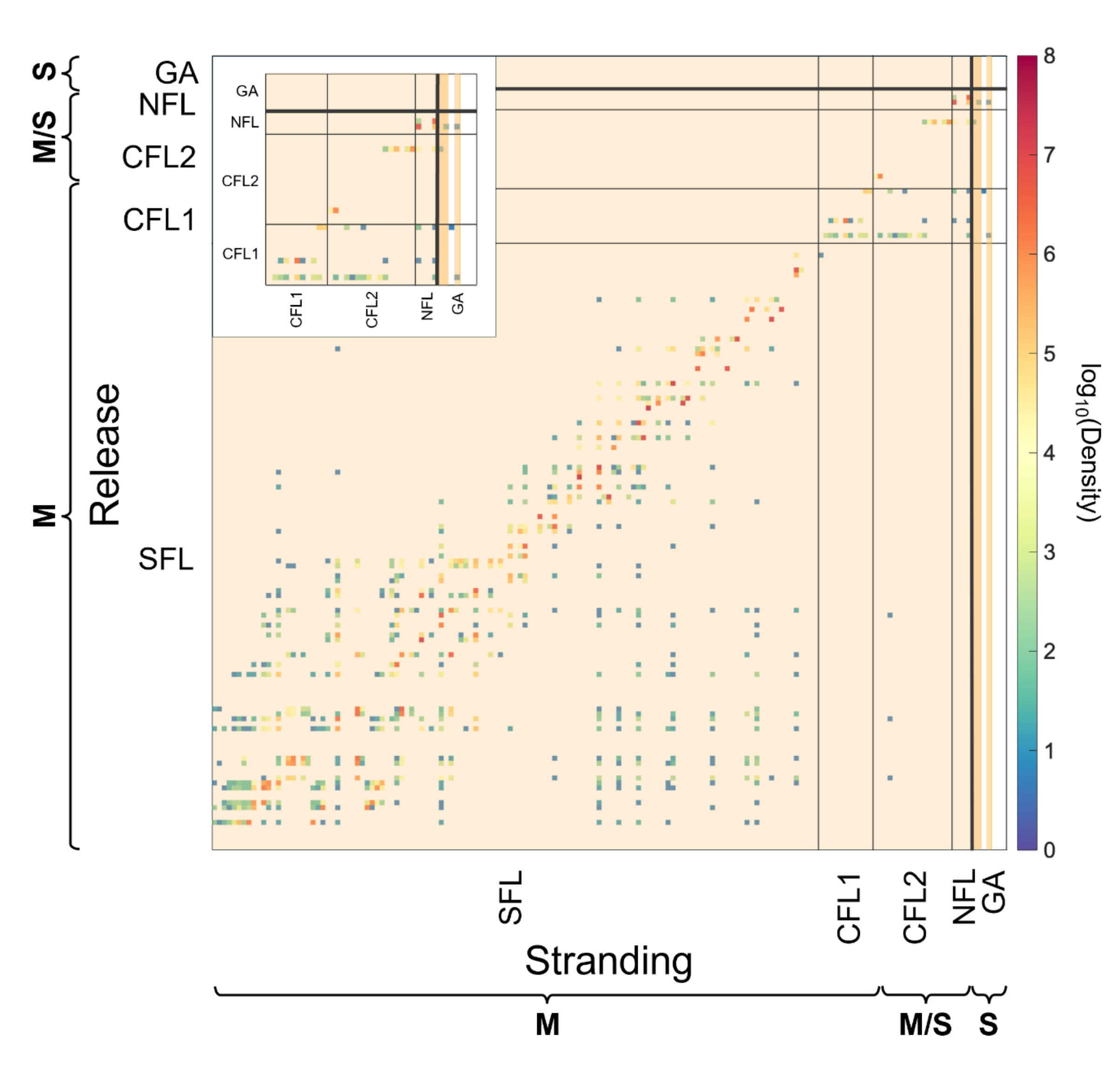

Fig. S14. Connectivity matrix between present mangrove populations (y-axis) and predicted stranding locations (x-axis). Subregion codes are defined as: **SFL**: Southeast Florida, **CFL1**: Central East Florida 1, **CFL2**: Central East Florida 2, **NFL**: Northeast Florida, and **GA**: Georgia (SI appendix, Fig. S3 for a geographic key to subregion codes). Lighter peach shading represents latitudes suitable under present climatic conditions. Darker peach shading represents latitudes identified as suitable under SSP3-7.0. No shading represents latitudes projected to be unsuitable based on climate. The thick horizontal black line marks the Florida-Georgia border, which is near the current range limit of mangroves. Curly brackets indicate the transition between present-day mangrove-dominated (**M**),

mangrove-saltmarsh ecotone-dominated (**M/S**), and saltmarsh-dominated latitudes (**S**), as described in Cavanaugh *et al*. (2019). Inset: zoomed-in view of the region near the current range limit, spanning CFL1 to GA. The connectivity matrix was generated using output from a Lagrangian particle-tracking model with a floating period of 6 months and a minimum floating period that consisted of a Monte Carlo simulation that generated random values between 1–5 days.

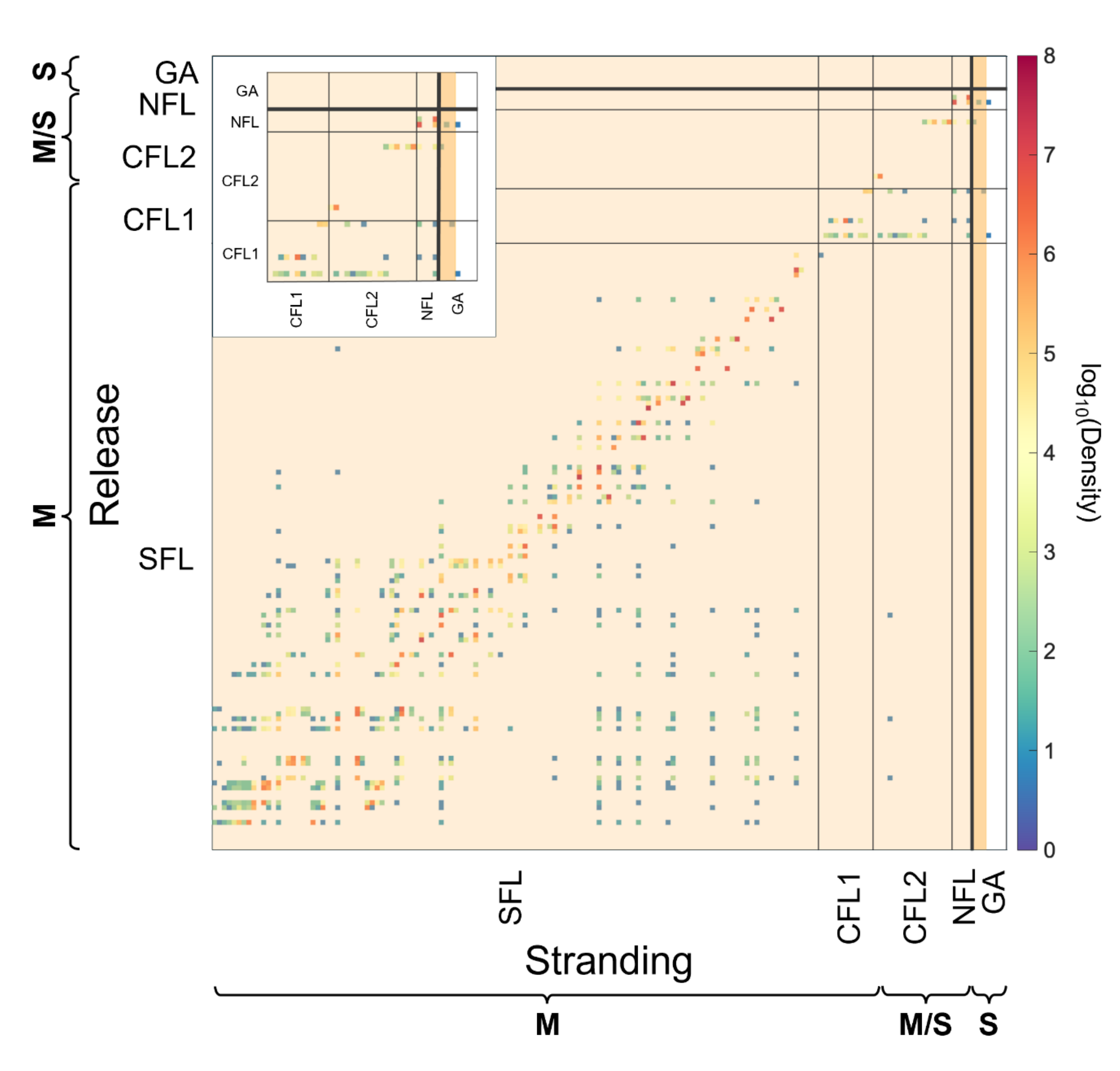

Fig. S15. Connectivity matrix between present mangrove populations (y-axis) and predicted stranding locations (x-axis). Subregion codes are defined as: **SFL**: Southeast Florida, **CFL1**: Central East Florida 1, **CFL2**: Central East Florida 2, **NFL**: Northeast Florida, and **GA**: Georgia (SI appendix, Fig. 3 for a geographic key to subregion codes). Lighter peach shading represents latitudes suitable under present climatic conditions. Darker peach shading represents latitudes identified as suitable under SSP5-8.5. No shading represents latitudes projected to be unsuitable based on climate. The thick horizontal black line marks the Florida-Georgia border, which is near the current range limit of mangroves. Curly brackets indicate the transition between present-day mangrove-dominated (**M**),

mangrove-saltmarsh ecotone-dominated (**M/S**), and saltmarsh-dominated latitudes (**S**), as described in Cavanaugh *et al*. (2019). Inset: zoomed-in view of the region near the current range limit, spanning CFL1 to GA. The connectivity matrix was generated using output from a Lagrangian particle-tracking model with a floating period of 6 months and a minimum floating period that consisted of a Monte Carlo simulation that generated random values between 1–5 days.

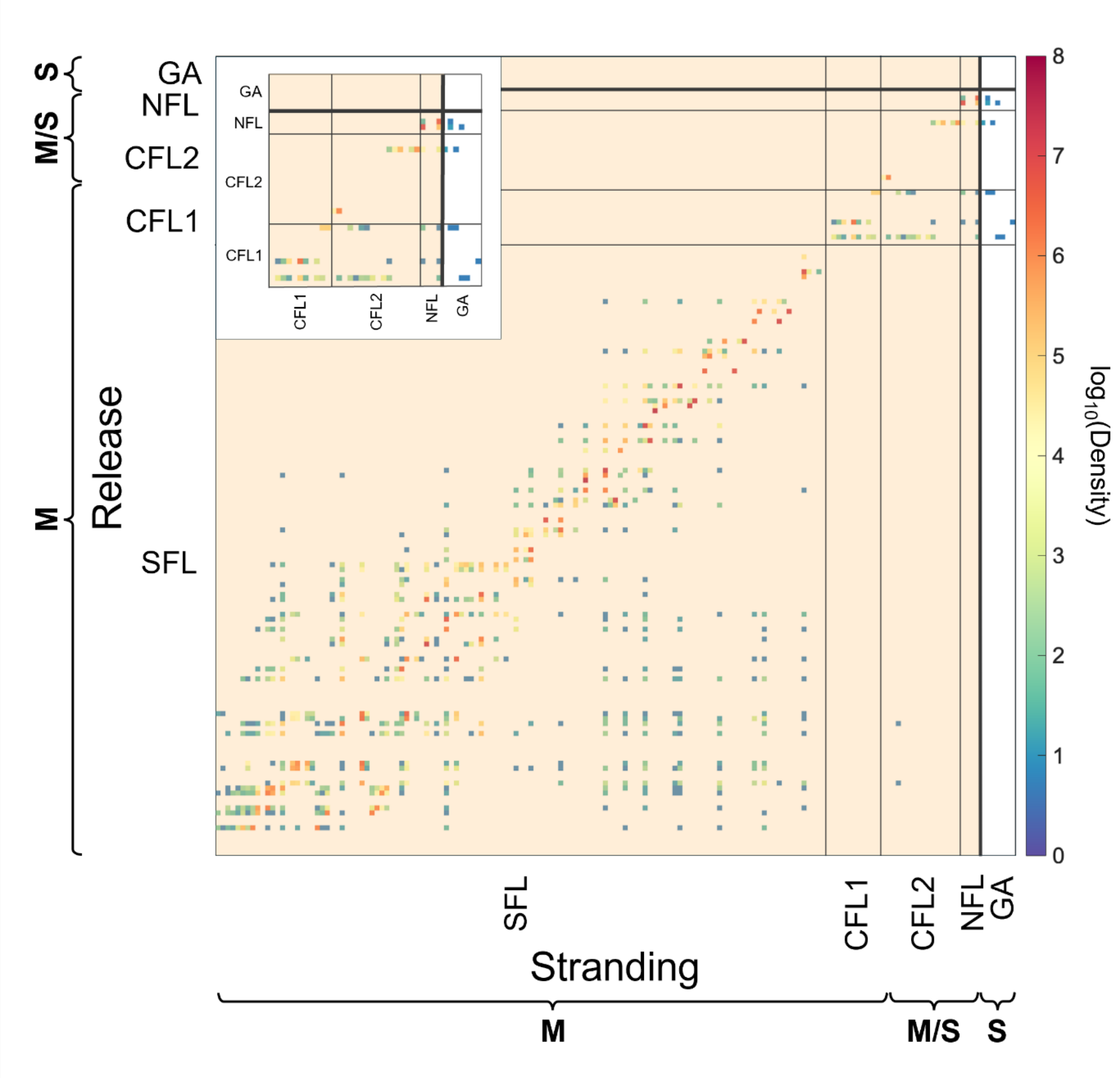

Fig. S16. Connectivity matrix between present mangrove populations (y-axis) and predicted stranding locations (x-axis). Subregion codes are defined as: **SFL**: Southeast Florida, **CFL1**: Central East Florida 1, **CFL2**: Central East Florida 2, **NFL**: Northeast Florida, and **GA**: Georgia (SI appendix, Fig. S3 for a geographic key to subregion codes). Lighter peach shading represents latitudes suitable under present climatic conditions. Darker peach shading represents latitudes identified as suitable under SSP1-2.6. No shading represents latitudes projected to be unsuitable based on climate. The thick horizontal black line marks the Florida-Georgia border, which is near the current range limit of mangroves. Curly brackets indicate the transition between present-day mangrove-dominated (**M**), mangrove-saltmarsh ecotone-dominated (**M/S**), and saltmarsh-dominated latitudes (**S**), as described

in Cavanaugh *et al*. (2019). Inset: zoomed-in view of the region near the current range limit, spanning CFL1 to GA. The connectivity matrix was generated using output from a Lagrangian particle-tracking model with a floating period of 12 months and a minimum floating period that consisted of a Monte Carlo simulation that generated random values between 1–5 days.

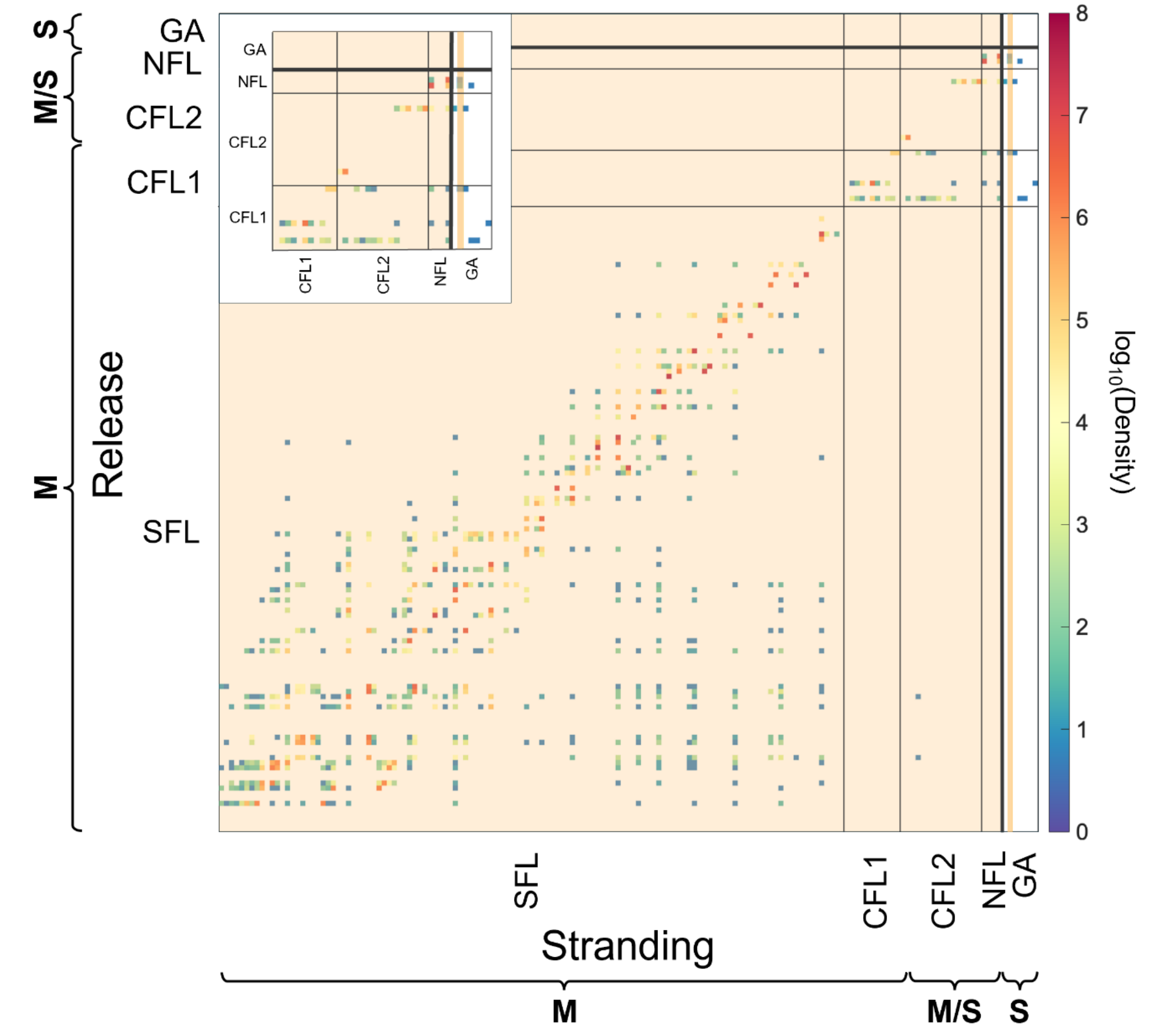

Fig. S17. Connectivity matrix between present mangrove populations (y-axis) and predicted stranding locations (x-axis). Subregion codes are defined as: **SFL**: Southeast Florida, **CFL1**: Central East Florida 1, **CFL2**: Central East Florida 2, **NFL**: Northeast Florida, and **GA**: Georgia (SI appendix, Fig. S3 for a geographic key to subregion codes). Lighter peach shading represents latitudes suitable under present climatic conditions. Darker peach shading represents latitudes identified as suitable under SSP2-4.5. No shading represents latitudes projected to be unsuitable based on climate. The thick horizontal black line marks the Florida-Georgia border, which is near the current range limit of mangroves. Curly brackets indicate the transition between present-day mangrove-dominated (**M**), mangrove-saltmarsh ecotone-dominated (**M/S**), and saltmarsh-dominated latitudes (**S**), as described

in Cavanaugh *et al*. (2019). Inset: zoomed-in view of the region near the current range limit, spanning CFL1 to GA. The connectivity matrix was generated using output from a Lagrangian particle-tracking model with a floating period of 12 months and a minimum floating period that consisted of a Monte Carlo simulation that generated random values between 1–5 days.

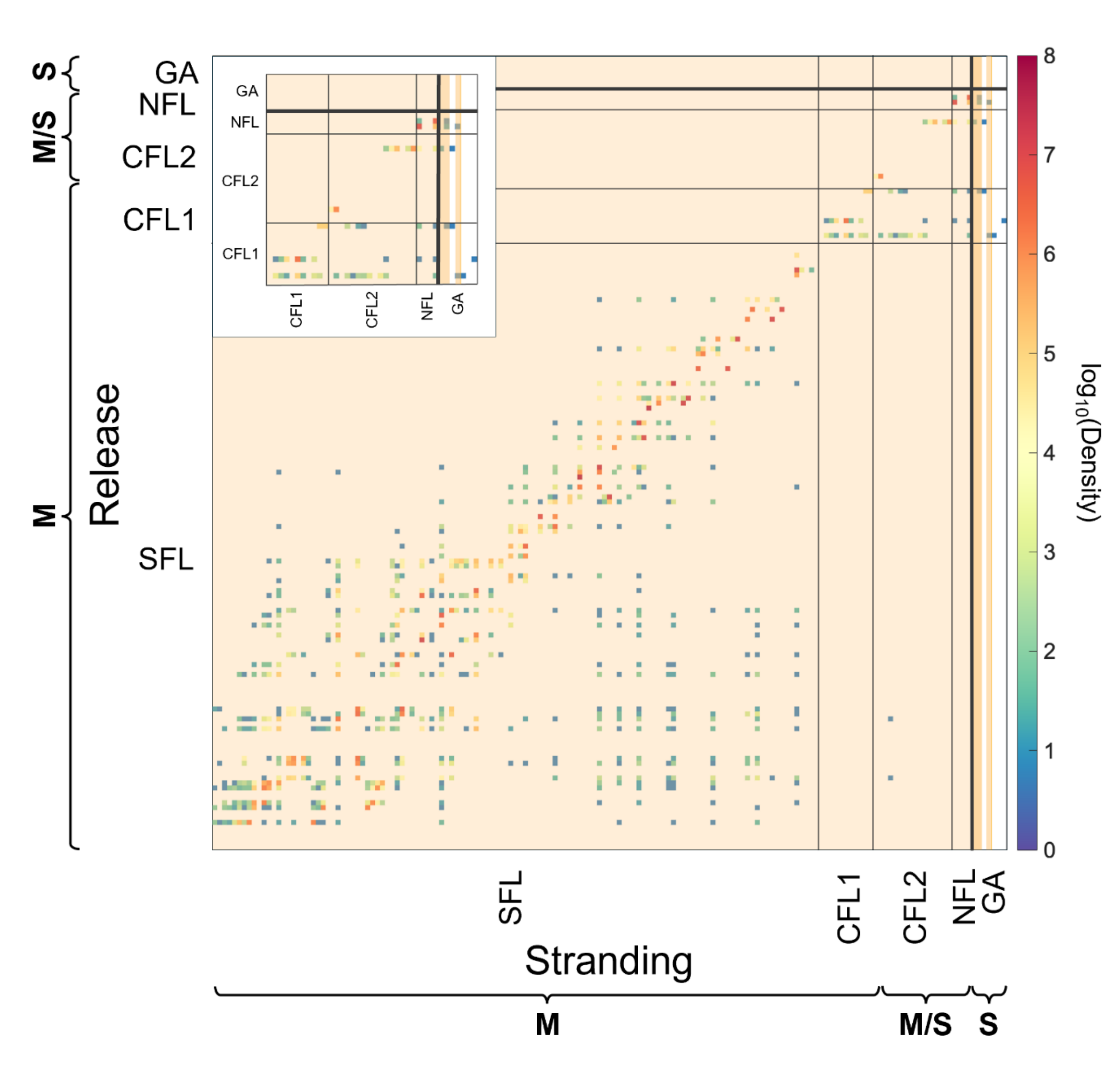

Fig. S18. Connectivity matrix between present mangrove populations (y-axis) and predicted stranding locations (x-axis). Subregion codes are defined as: **SFL**: Southeast Florida, **CFL1**: Central East Florida 1, **CFL2**: Central East Florida 2, **NFL**: Northeast Florida, and **GA**: Georgia (SI appendix, Fig. S3 for a geographic key to subregion codes). Lighter peach shading represents latitudes suitable under present climatic conditions. Darker peach shading represents latitudes identified as suitable under SSP3-7.0. No shading represents latitudes projected to be unsuitable based on climate. The thick horizontal black line marks the Florida-Georgia border, which is near the current range limit of mangroves. Curly brackets indicate the transition between present-day mangrove-dominated (**M**),

mangrove-saltmarsh ecotone-dominated (**M/S**), and saltmarsh-dominated latitudes (**S**), as described in Cavanaugh *et al*. (2019). Inset: zoomed-in view of the region near the current range limit, spanning CFL1 to GA. The connectivity matrix was generated using output from a Lagrangian particle-tracking model with a floating period of 12 months and a minimum floating period that consisted of a Monte Carlo simulation that generated random values between 1–5 days.

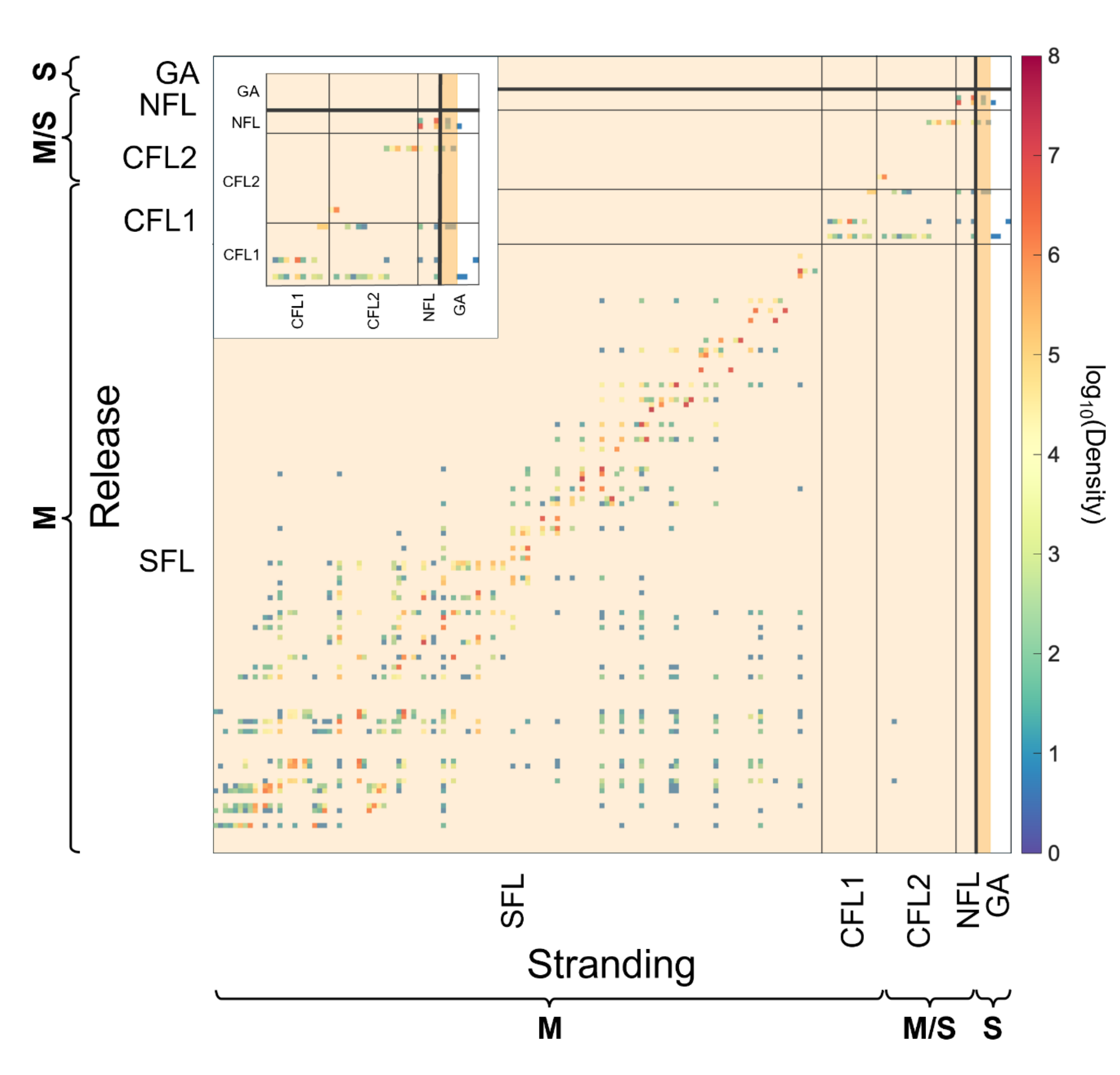

Fig. S19. Connectivity matrix between present mangrove populations (y-axis) and predicted stranding locations (x-axis). Subregion codes are defined as: **SFL**: Southeast Florida, **CFL1**: Central East Florida 1, **CFL2**: Central East Florida 2, **NFL**: Northeast Florida, and **GA**: Georgia (SI appendix, Fig. S3 for a geographic key to subregion codes). Lighter peach shading represents latitudes suitable under present climatic conditions. Darker peach shading represents latitudes identified as suitable under SSP5-8.5. No shading represents latitudes projected to be unsuitable based on climate. The thick horizontal black line marks the Florida-Georgia border, which is near the current range limit of mangroves. Curly brackets indicate the transition between present-day mangrove-dominated (**M**),

mangrove-saltmarsh ecotone-dominated (**M/S**), and saltmarsh-dominated latitudes (**S**), as described in Cavanaugh *et al*. (2019). Inset: zoomed-in view of the region near the current range limit, spanning CFL1 to GA. The connectivity matrix was generated using output from a Lagrangian particle-tracking model with a floating period of 12 months and a minimum floating period that consisted of a Monte Carlo simulation that generated random values between 1–5 days.

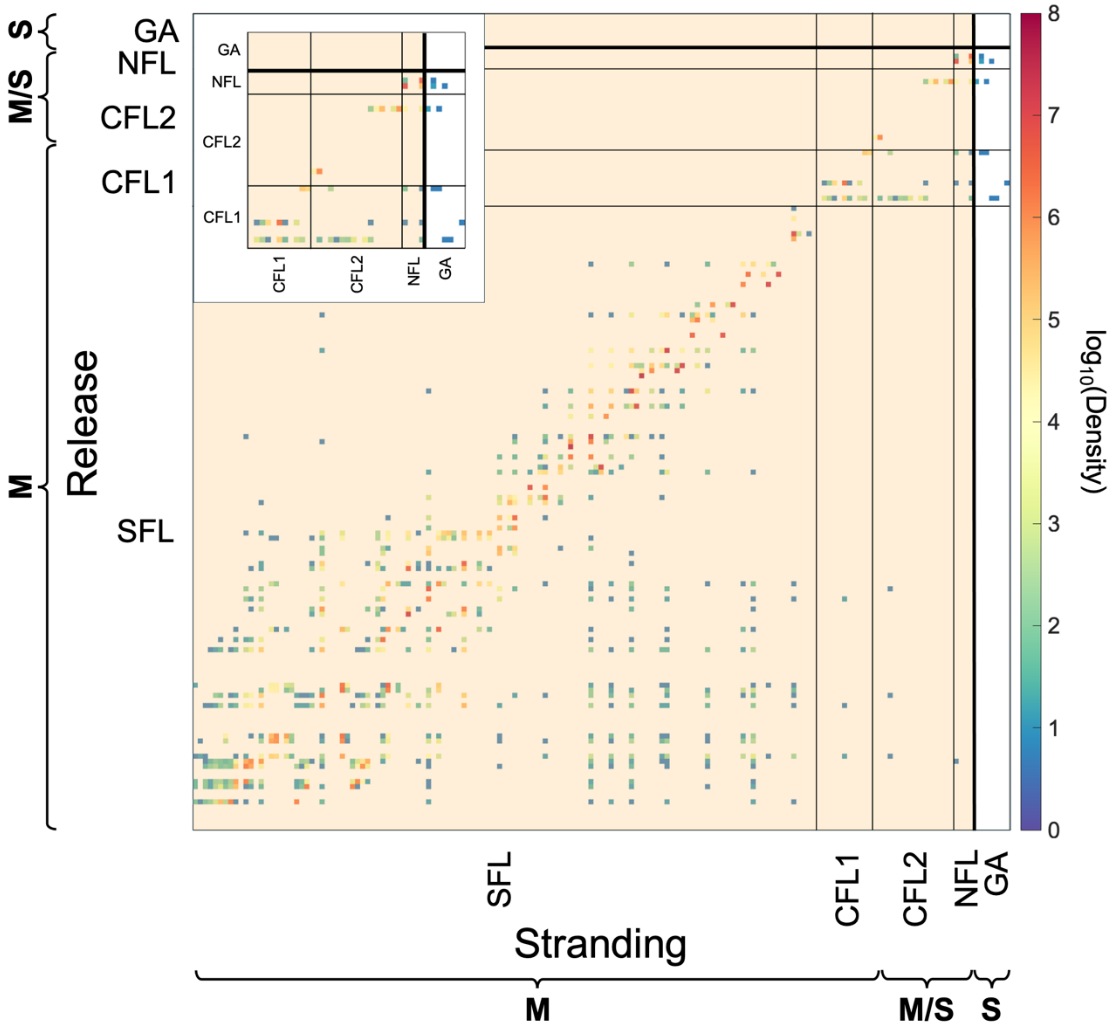

Fig. S20. Connectivity matrix between present mangrove populations (y-axis) and predicted stranding locations (x-axis). Subregion codes are defined as: **SFL**: Southeast Florida, **CFL1**: Central East Florida 1, **CFL2**: Central East Florida 2, **NFL**: Northeast Florida, and **GA**: Georgia (SI appendix, Fig. S3 for a geographic key to subregion codes). Lighter peach shading represents latitudes suitable under present climatic conditions. Darker peach shading represents latitudes identified as suitable under SSP1-2.6. No shading represents latitudes projected to be unsuitable based on climate. The thick horizontal black line marks the Florida-Georgia border, which is near the current range limit of mangroves. Curly brackets indicate the transition between present-day mangrove-dominated (**M**), mangrove-saltmarsh ecotone-dominated (**M/S**), and saltmarsh-dominated latitudes (**S**), as described

in Cavanaugh *et al*. (2019). Inset: zoomed-in view of the region near the current range limit, spanning CFL1 to GA. The connectivity matrix was generated using output from a Lagrangian particle-tracking model with a floating period of 17 months and a minimum floating period that consisted of a Monte Carlo simulation that generated random values between 1–5 days.

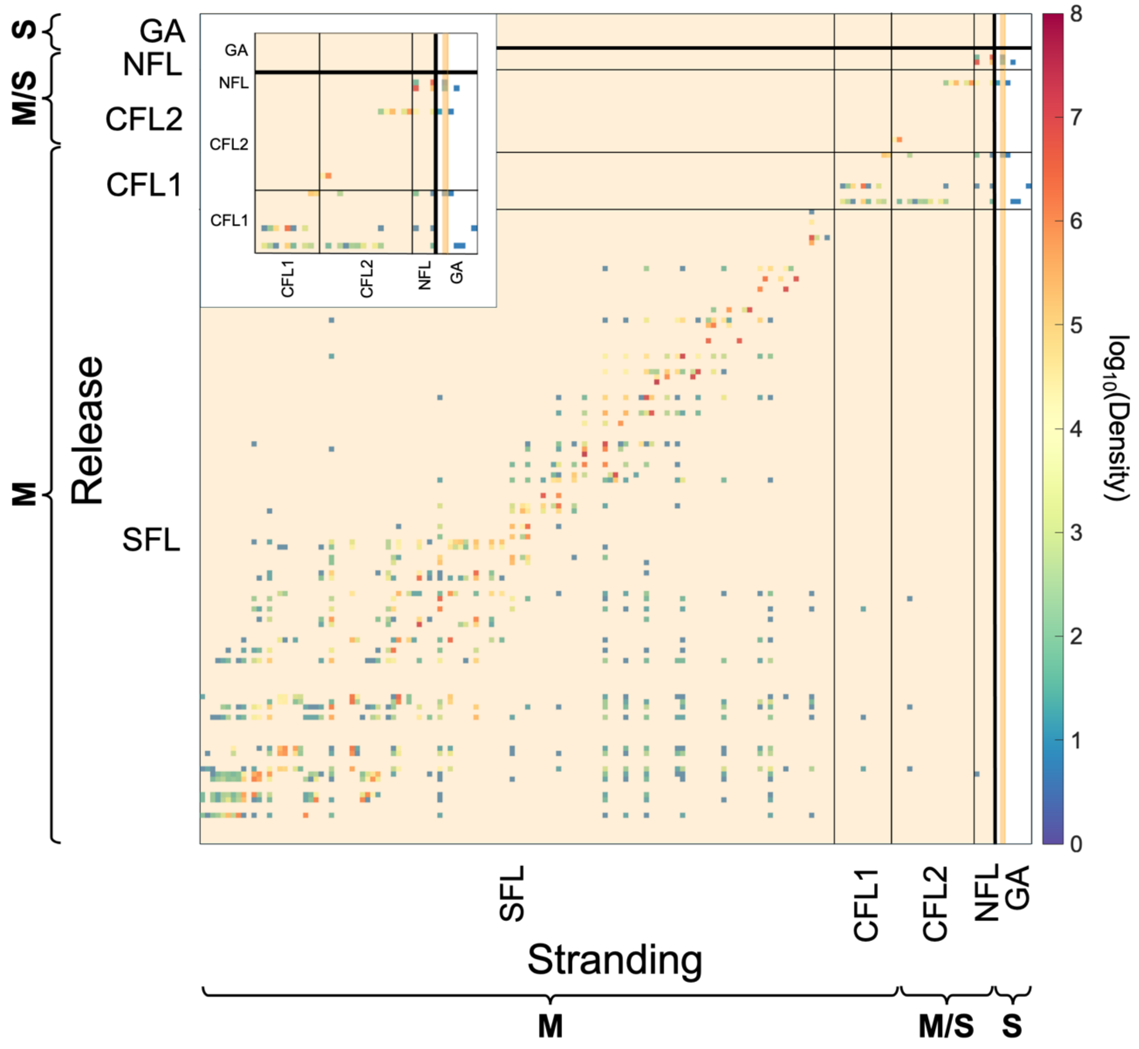

Fig. S21. Connectivity matrix between present mangrove populations (y-axis) and predicted stranding locations (x-axis). Subregion codes are defined as: **SFL**: Southeast Florida, **CFL1**: Central East Florida 1, **CFL2**: Central East Florida 2, **NFL**: Northeast Florida, and **GA**: Georgia (SI appendix, Fig. S3 for a geographic key to subregion codes). Lighter peach shading represents latitudes suitable under present climatic conditions. Darker peach shading represents latitudes identified as suitable under SSP2-4.5. No shading represents latitudes projected to be unsuitable based on climate. The thick horizontal black line marks the Florida-Georgia border, which is near the current range limit of mangroves. Curly brackets indicate the transition between present-day mangrove-dominated (**M**), mangrove-saltmarsh ecotone-dominated (**M/S**), and saltmarsh-dominated latitudes (**S**), as described

in Cavanaugh *et al*. (2019). Inset: zoomed-in view of the region near the current range limit, spanning CFL1 to GA. The connectivity matrix was generated using output from a Lagrangian particle-tracking model with a floating period of 17 months and a minimum floating period that consisted of a Monte Carlo simulation that generated random values between 1–5 days.

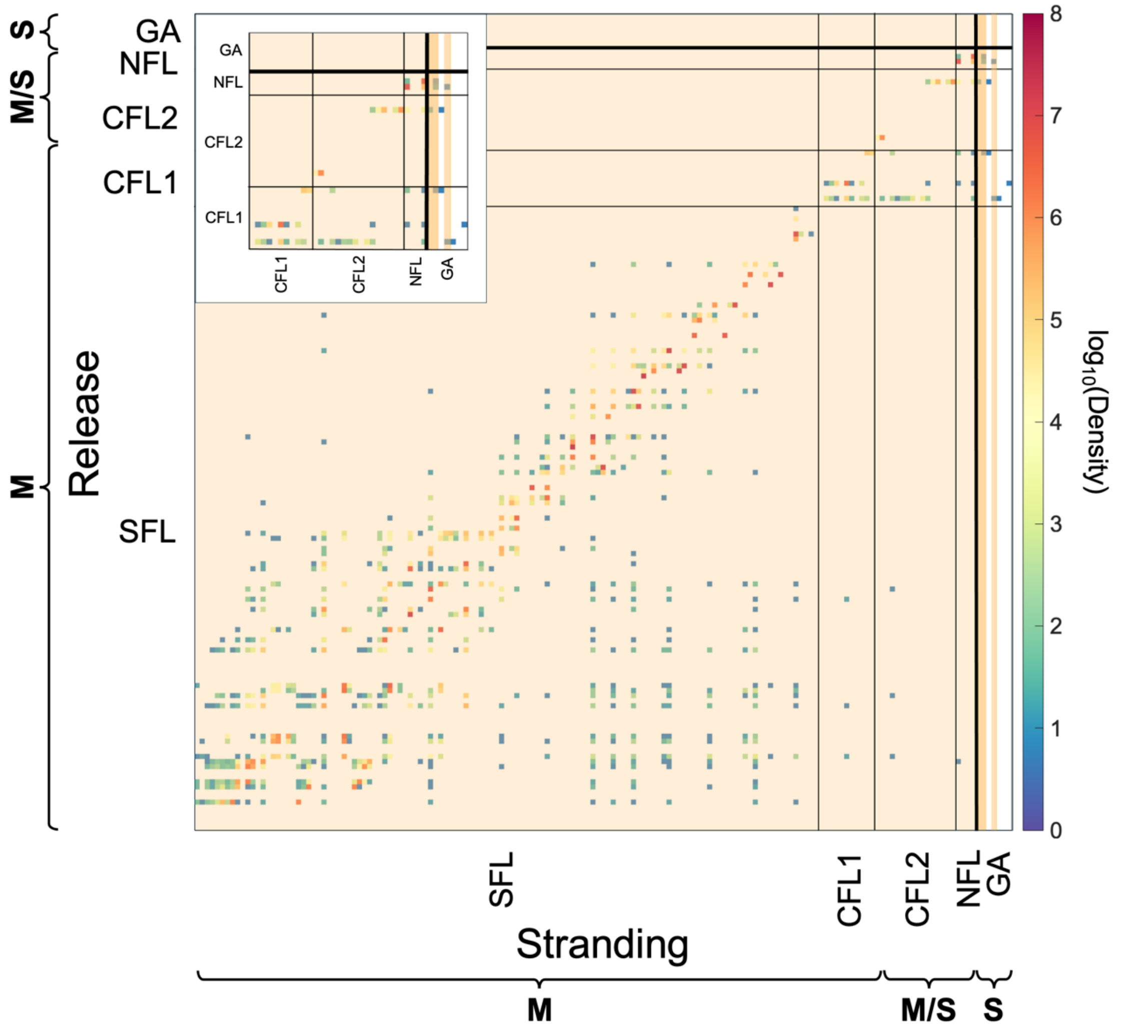

Fig. S22. Connectivity matrix between present mangrove populations (y-axis) and predicted stranding locations (x-axis). Subregion codes are defined as: **SFL**: Southeast Florida, **CFL1**: Central East Florida 1, **CFL2**: Central East Florida 2, **NFL**: Northeast Florida, and **GA**: Georgia (SI appendix, Fig. S3 for a geographic key to subregion codes). Lighter peach shading represents latitudes suitable under present climatic conditions. Darker peach shading represents latitudes identified as suitable under SSP3-7.0. No shading represents latitudes projected to be unsuitable based on climate. The thick horizontal black line marks the Florida-Georgia border, which is near the current range limit of mangroves. Curly brackets indicate the transition between present-day mangrove-dominated (**M**), mangrove-saltmarsh ecotone-dominated (**M/S**), and saltmarsh-dominated latitudes (**S**), as described in Cavanaugh *et al*. (2019). Inset: Zoomed-in view of the region near the current range limit, spanning

CFL1 to GA. The connectivity matrix was generated using output from a Lagrangian particle-tracking model with a floating period of 17 months and a minimum floating period that consisted of a Monte Carlo simulation that generated random values between 1–5 days.

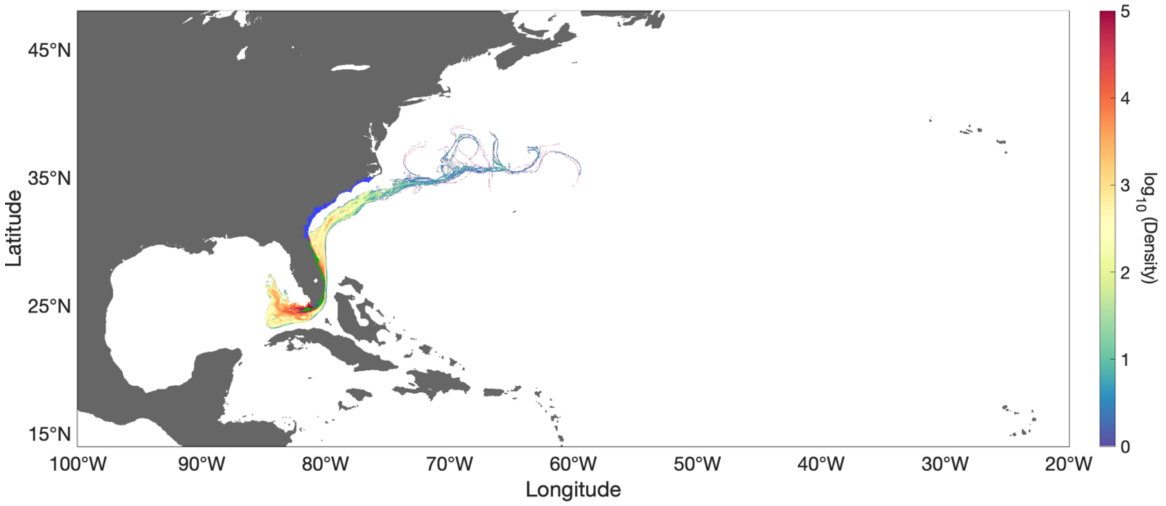

Fig. S23. Simulated trajectory density map of mangrove propagules released along the eastern coast of Florida. Propagules were released hourly over a three-month period (August–October) and allowed a maximum floating period of 1 month. Dispersal trajectories were aggregated on a 1/24° × 1/24° grid. Green and blue points along the Atlantic coast of Florida indicate present-day mangrove and salt marsh occurrence, respectively, with particles released from present-day mangrove locations (green). Map lines delineate study areas and do not necessarily depict accepted national boundaries.

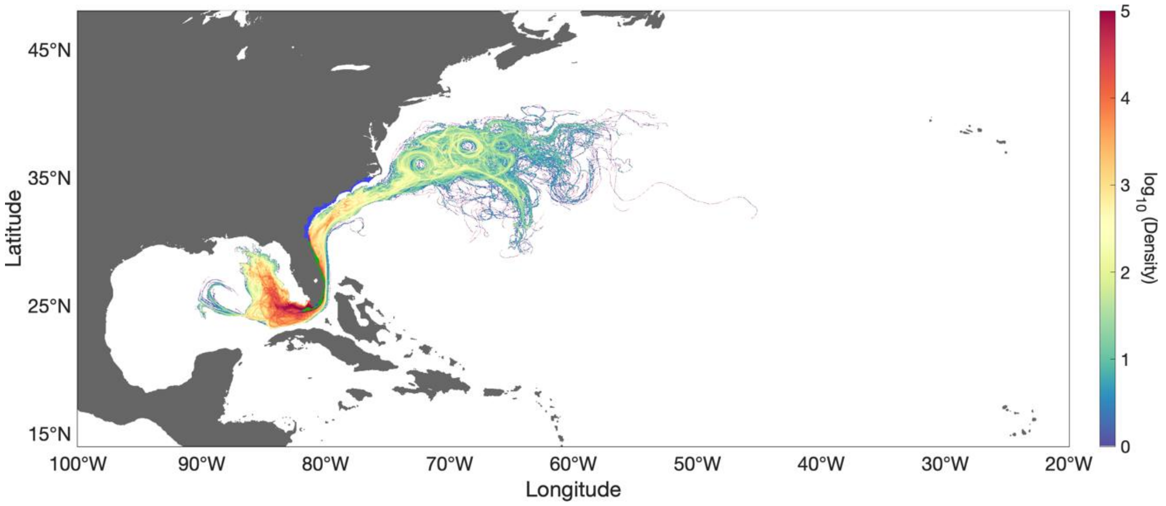

Fig. S24. Simulated trajectory density map of mangrove propagules released along the eastern coast of Florida. Propagules were released hourly over a three-month period (August–October) and allowed a maximum floating period of 3 months. Dispersal trajectories were aggregated on a 1/24° × 1/24° grid. Green and blue points along the Atlantic coast of Florida indicate present-day mangrove and salt marsh occurrence, respectively, with particles released from present-day mangrove locations (green). Map lines delineate study areas and do not necessarily depict accepted national boundaries.

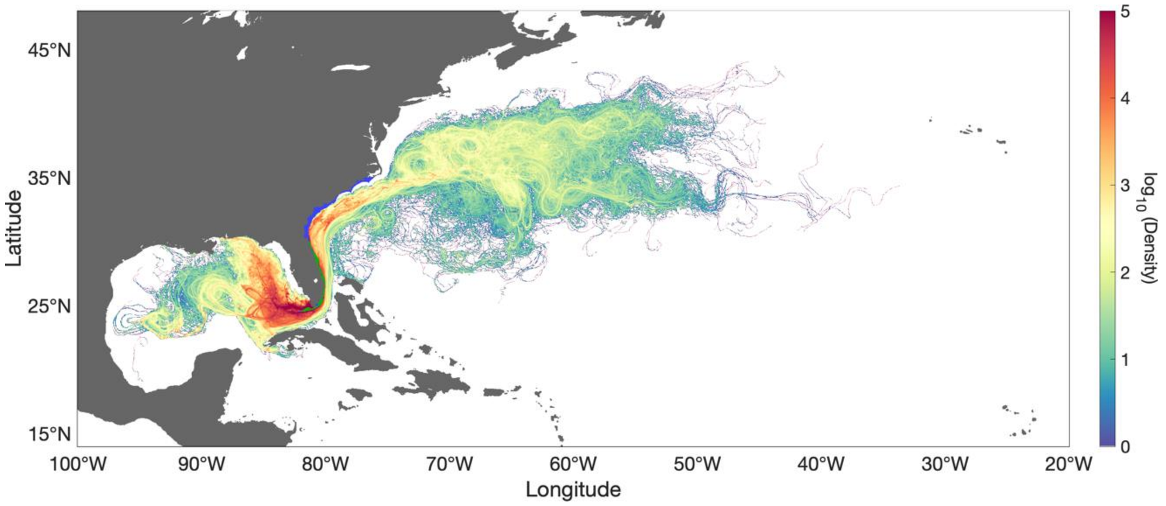

Fig. S25. Simulated trajectory density map of mangrove propagules released along the eastern coast of Florida. Propagules were released hourly over a three-month period (August–October) and allowed a maximum floating period of 6 months. Dispersal trajectories were aggregated on a 1/24° × 1/24° grid. Green and blue points along the Atlantic coast of Florida indicate present-day mangrove and salt marsh occurrence, respectively, with particles released from present-day mangrove locations (green). Map lines delineate study areas and do not necessarily depict accepted national boundaries.

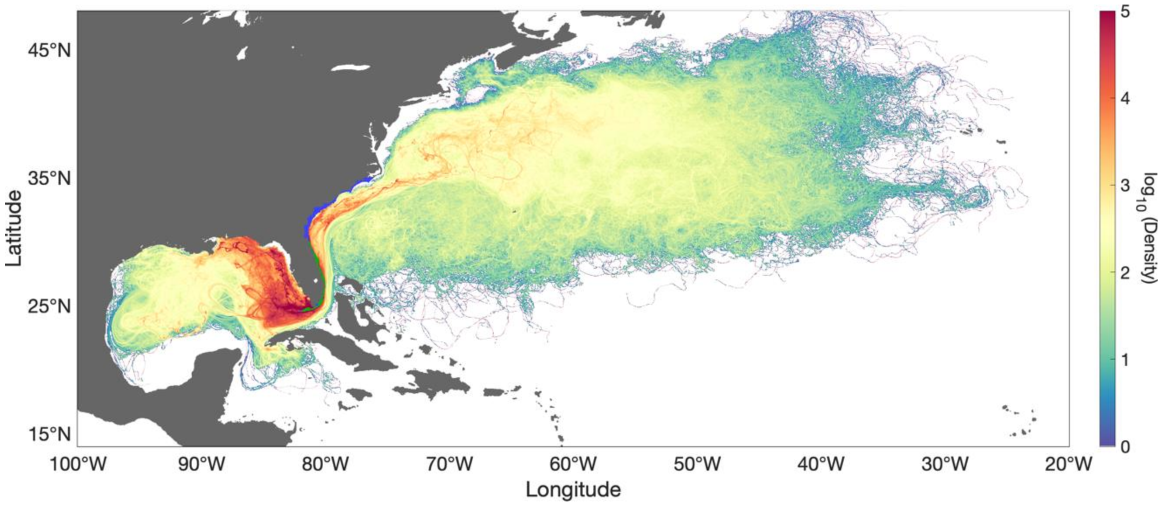

Fig. S26. Simulated trajectory density map of mangrove propagules released along the eastern coast of Florida. Propagules were released hourly over a three-month period (August–October) and allowed a maximum floating period of 12 months. Dispersal trajectories were aggregated on a 1/24° × 1/24° grid. Green and blue points along the Atlantic coast of Florida indicate present-day mangrove and salt marsh occurrence, respectively, with particles released from present-day mangrove locations (green). Map lines delineate study areas and do not necessarily depict accepted national boundaries.

SI References

1. Florida Department of Environmental Protection, Data from "Statewide Land Use Land Cover". Available at https://geodata.dep.state.fl.us/datasets/FDEP::statewide-land-use-land-cover/about. Deposited 2022.

2. Cavanaugh, K. C. *et al.* 2019. Climate-driven regime shifts in a mangrove–salt marsh ecotone over the past 250 years. *Proceedings of the National Academy of Sciences U.S.A.,* **116**(43), 21602–21608. https://doi.org/10.1073/pnas.1902181116.

3. UNEP-WCMC, Data from "Ocean+ Habitats". Available at https://data.unep-wcmc.org/datasets/43. Deposited 2025.
